# Enhancing Tsuji–Trost deallylation in living cells with an internal-nucleophile coumarin-based probe†

**DOI:** 10.1039/d3ra08938j

**Published:** 2024-02-13

**Authors:** Yonghua Tan, François Pierrard, Raphaël Frédérick, Olivier Riant

**Affiliations:** a Institute of Condensed Matter and Nanosciences (IMCN), Université catholique de Louvain Louvain-la-Neuve 1348 Belgium olivier.riant@uclouvain.be; b Louvain Drug Research Institute (LDRI), Université catholique de Louvain Brussels B-1200 Belgium raphael.frederick@uclouvain.be

## Abstract

In recent years, bioorthogonal uncaging reactions have been developed to proceed efficiently under physiological conditions. However, limited progress has been made in the development of protecting groups combining stability under physiological settings with the ability to be quickly removed *via* bioorthogonal catalysis. Herein, we present a new water-soluble coumarin-derived probe bearing an internal nucleophilic group capable of promoting Tsuji–Trost deallylation under palladium catalysis. This probe can be cleaved by a bioorthogonal palladium complex at a faster rate than the traditional probe, namely *N*-Alloc-7-amino-4-methylcoumarin. As the deallylation process proved to be efficient in mammalian cells, we envision that this probe may find applications in chemical biology, bioengineering, and medicine.

## Abstract 2

The grafting of a diisopropylaminobenzyl substituent onto an *N*-Alloc protecting group significantly accelerates Tsuji–Trost deallylation, enabling intramolecular capture of the π-allylpalladium intermediate.

## Graphical abstract

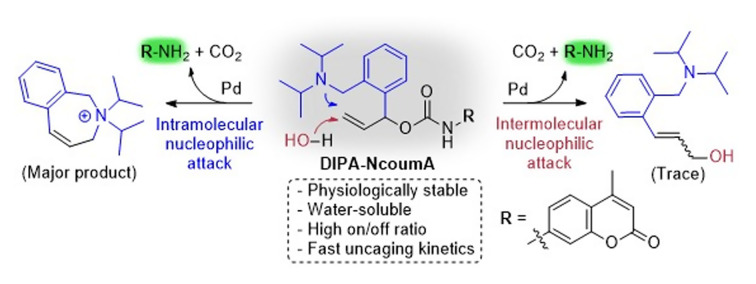

## Introduction

**Scheme 1:**
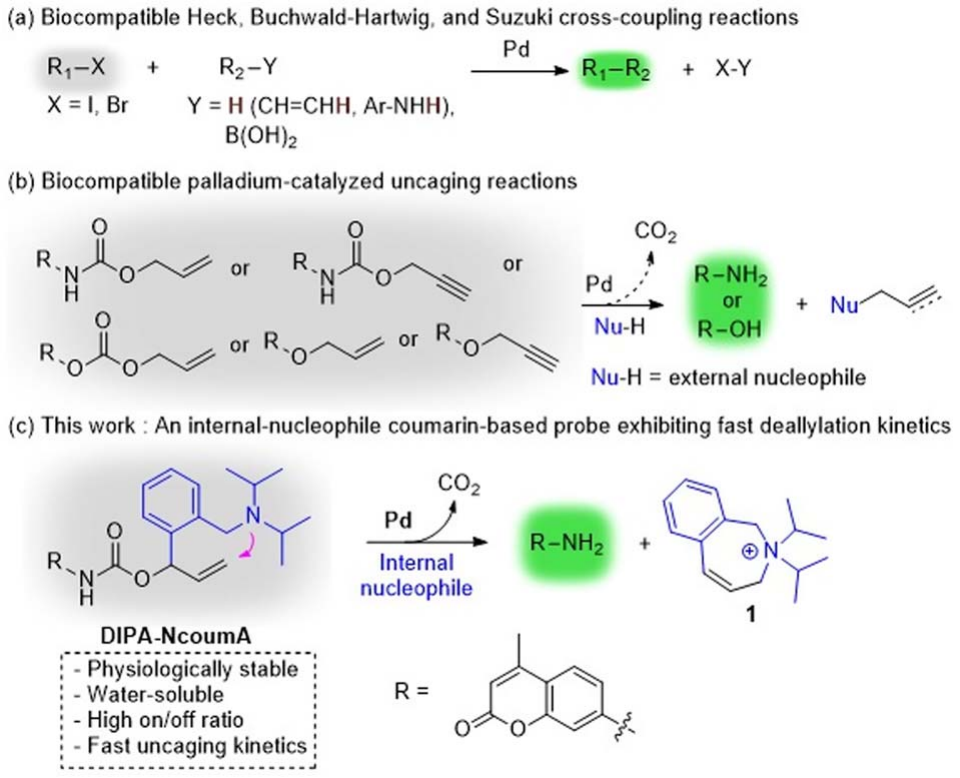
Generation of fluorescent compounds *via* biocompatible palladium-catalyzed reactions.

**Scheme 2:**
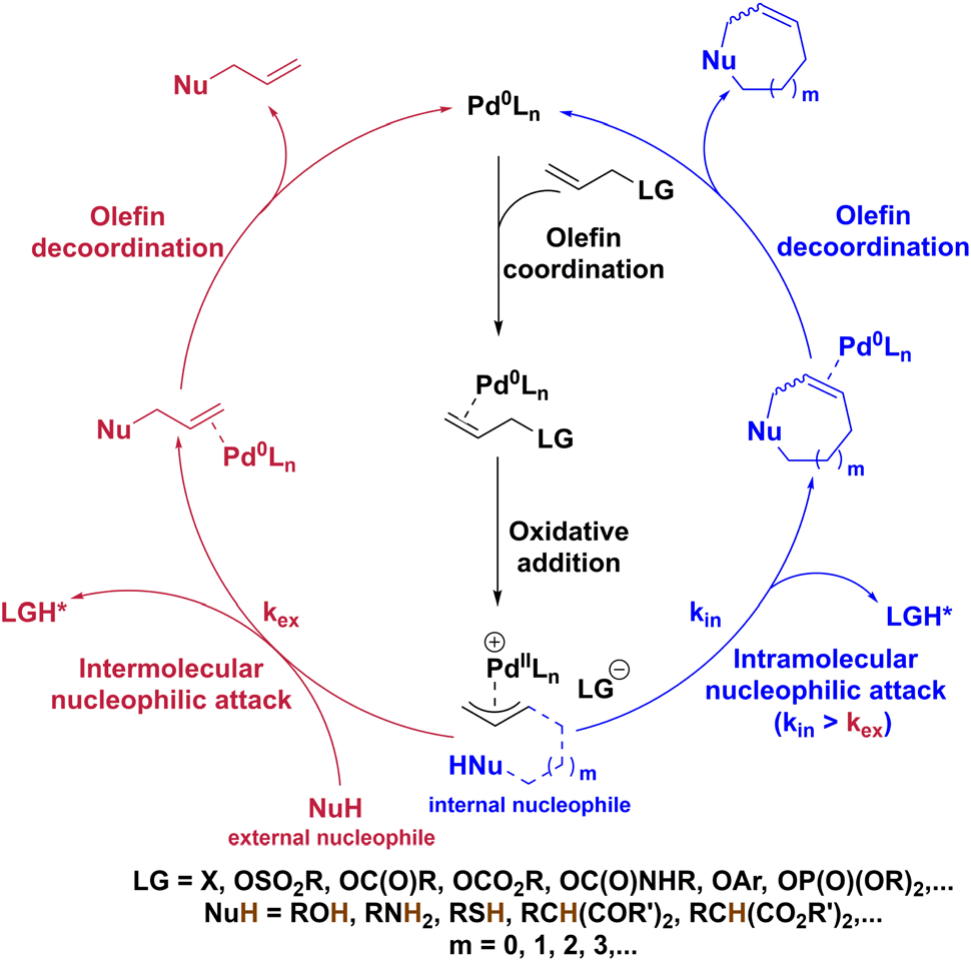
Catalytic cycle of intermolecular (left) and intramolecular (right) Tsuji–Trost reactions. LG: leaving group; X: halogen; NuH: (hetero)nucleophile. * If the counter anion LG^−^ (corresponding to the leaving group or obtained after its decarboxylation) is sufficiently basic, it can deprotonate the nucleophile itself. If not, another basic species must be present to allow the deprotonation of the nucleophile.

Bioorthogonal chemistry has emerged as a promising field in chemical biology, offering versatile tools for studying and manipulating biological systems. Indeed, reactions such as copper-catalyzed azide–alkyne cycloaddition (CuAAC),^1–6^ Staudinger ligation,^7–10^ strain-promoted azide–alkyne cycloaddition (SPAAC),^11,12^ and inverse electron-demand Diels–Alder reaction (IEDDA),^13–17^ have been characterized by their compatibility with living systems, allowing specific chemical transformations to occur without interfering with native biochemical processes.^18–21^ Moreover, transition metals, especially palladium, have sparked significant interest in the development of imaging probes and prodrugs, as these metals can catalyze biocompatible cross-coupling and uncaging reactions, leading to the formation of a compound of interest.^22–24^ In 2017, the Weissleder group reported that a fluorescent coumarin product was formed in HT1080 cells *via* an intramolecular Heck reaction catalyzed by a Pd(0) complex.^25^ Also, in 2019, the Neto group transformed nonfluorescent compounds into 2,1,3-benzothiadiazole-derived fluorescent probes through *in cellulo* Suzuki and Buchwald–Hartwig cross-coupling reactions (Scheme 1a).^26^ Alongside cross-coupling reactions, palladium-promoted uncaging reactions are even more popular for generating molecules of interest in biological settings. In 2011, the Bradley group demonstrated the release of a fluorescent rhodamine derivative through Pd(0)-catalyzed removal of an *N*-Alloc moiety within cellular environments.^27^ The same year, the Liu group pioneered a naphthalimide-based fluorescent probe, capitalizing on Pd(0)-catalyzed *N*-Alloc cleavage under mild conditions.^28^ Subsequently, uncaging of *N*-Alloc-protected compounds have been applied widely in the field of fluorescent probes.^25,29–37^ In this uncaging chemistry context, other diverse protecting groups have been used, including *N*-propargyloxycarbonyl,^38–43^*O*-allyloxycarbonyl,^44–47^*O*-allyl,^48–52^ and *O*-propargyl^42,53–55^ groups (Scheme 1b). All these chemical moieties can indeed reveal a free amine or free alcohol following palladium-catalyzed Tsuji–Trost deallylation or depropargylation. This highlights the importance and potency of the Tsuji–Trost reaction in bioorthogonal uncaging chemistry.^56^ Typically, the Tsuji–Trost reaction occurs when a nucleophile traps a π-allylpalladium electrophile, which is obtained by coordination of an olefinic substrate to a Pd(0) species followed by oxidative addition. Kinetics of olefin coordination and oxidative addition can be tuned by the properties of ligands, such as bite angles, and electronic and steric factors.^34,52,57–59^ Regarding the nucleophilic attack, this usually involves an external nucleophile, which, in biological media, can be a cysteine or tyrosine residue.^60–63^ This step can be a slower process, especially if the nucleophile is not readily available close to the π-allylpalladium species. Conversely, intramolecular trapping of π-allylpalladium cations allows for particularly fast reactions (Scheme 2).^64^

Herein, we present an original probe, namely diisopropylaminobenzyl-*N*-Alloc-7-amino-4-methylcoumarin (DIPA-NcoumA), that can be subjected to intramolecular Tsuji–Trost deallylation in living cells (Scheme 1c). This probe is cleaved by a bioorthogonal palladium complex at a faster rate than the traditional probe *N*-Alloc-7-amino-4-methylcoumarin (NcoumA). DIPA-NcoumA also shows an improvement in the on/off ratio, which can make fluorescence measurements easier. Lastly, we propose a plausible mechanism for the uncaging of 7-amino-4-methylcoumarin (Ncoum) from DIPA-NcoumA, thanks to the identification of the key cyclic product 1. We are confident that this versatile probe holds significant potential for widespread applications, notably in chemical biology.

## Results and discussion

**Fig. 1:**
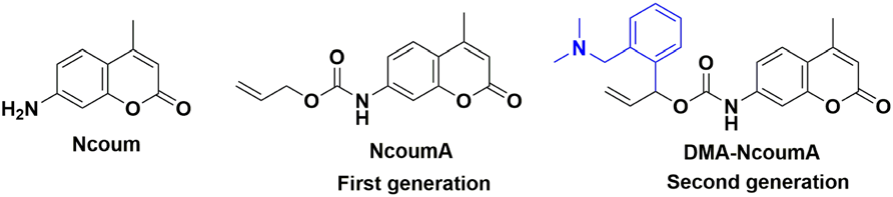
First- and second-generation caged Ncoum probes.

NcoumA is a very common caged probe in model reactions for optimizing conditions in bioorthogonal chemistry.^33–35,65,66^ Indeed, it can release the fluorescent compound Ncoum following a deallylation reaction. However, its poor water solubility requires the use of 10% DMSO^33^ or 0.05% Tween 20.^67^ Moreover, its low on/off ratio decreases the sensitivity of fluorescence-based analytical methods. In an effort to potentially address these issues, we aimed to design a new caged probe. Building upon the general mechanism of the Tsuji–Trost reaction, our main strategy was to enhance the deallylation kinetics by incorporating an internal nucleophilic group near the allyl carbamate moiety of NcoumA. Considering NcoumA as the first-generation probe, we synthesized a second-generation caged probe, dimethylaminobenzyl-NcoumA (DMA-NcoumA), whose structure includes an additional nucleophilic dimethylamino moiety in close proximity to the allyl carbamate (Fig. 1). This group was also supposed to increase the water solubility of the molecule.

**Fig. 2:**
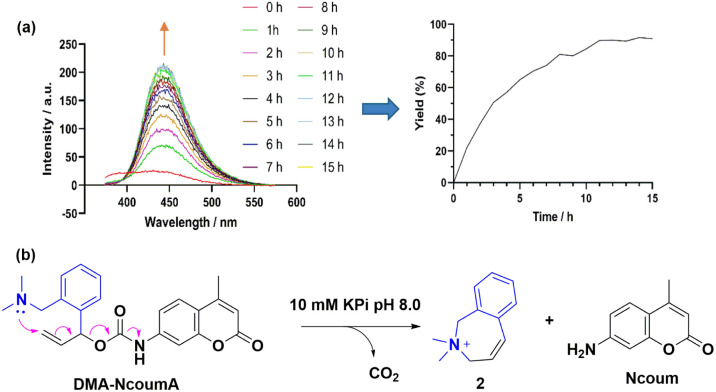
(a) Stability study of DMA-NcoumA (6 μM) in 1% DMSO in KPi (10 mM, pH 8.0) for 15 h at 37 °C, *λ*_ex_ = 342 nm, *λ*_em_ = 375–600 nm. (b) Proposed degradation mechanism.

Having the DMA-NcoumA probe in our hands, we evaluated its stability in conditions similar to physiological settings, namely in 10 mM potassium phosphate buffer (KPi, pH 8.0). Surprisingly, the fluorescence intensity (*λ*_ex_ = 342 nm, *λ*_em_ = 440 nm) increased over time in the absence of any palladium catalysts (Fig. 2a). By converting the fluorescence intensity at *λ*_em_ 440 nm into a kinetic curve, it became apparent that the DMA-NcoumA probe exhibited instability under the conditions used, resulting in the spontaneous release of the fluorescent compound Ncoum. We speculated that the nucleophilic dimethylamino group could attack the carbon–carbon double bond of the allyl carbamate moiety, leading to the liberation of CO_2_, Ncoum, and a cyclic quaternary ammonium 2 (Fig. 2b). ^1^H-NMR analysis of DMA-NcoumA was performed in DMSO-*d*_6_ for 1 h at room temperature, which showed signals corresponding to Ncoum (Fig. S2†). Besides, the formation of compound 2 was confirmed by LC-MS analysis (Fig. S3†). These findings corroborated the intrinsic instability of DMA-NcoumA under the given conditions.

**Fig. 3:**
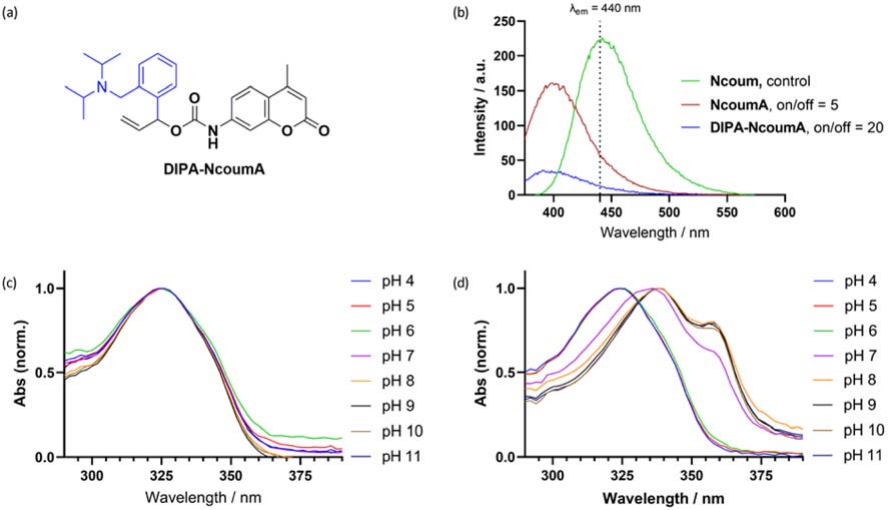
(a) Structure of DIPA-NcoumA. (b) Comparison of on/off ratios between NcoumA and DIPA-NcoumA. Conditions: Ncoum, NcoumA, and DIPA-NcoumA (6 μM) in 1% DMSO in KPi (10 mM, pH 8.0) *λ*_ex_ = 342 nm, *λ*_em_ = 375–600 nm (5% DMSO was necessary to dissolve NcoumA). UV absorption spectra of NcoumA (c) and DIPA-NcoumA (d) in aqueous buffers of varying pH (from 4 to 11). Absorption spectra were normalized at the maximal intensity of the main band.

Given the physiological instability of DMA-NcoumA, we embarked on the development of a third-generation caged probe. By replacing the dimethylamino moiety with a sterically hindered diisopropylamino group, we anticipated that the nearby vinyl group would be less prone to intramolecular nucleophilic attack, which led to the design of DIPA-NcoumA (Fig. 3a). We first conducted a stability study in 10 mM KPi (pH 8.0). Interestingly, we did not observe any changes in fluorescence intensity at *λ*_em_ 440 nm, suggesting that the diisopropylamino group did not engage in an intramolecular nucleophilic attack on the nearby vinyl group (Fig. S4a†). Moreover, LC-MS analysis did not reveal the appearance of Ncoum or a potential cyclic degradation product 1 (Fig. S4b†). ^1^H-NMR analysis of DIPA-NcoumA was also performed in DMSO-*d*_6_ for 24 h at room temperature, which did not show any signs of degradation (Fig. S5†). Based on these findings, we concluded that the introduction of the diisopropylamino group successfully prevented unwanted degradation, allowing the third-generation caged probe DIPA-NcoumA to be stable at pH 8.0.

Subsequently, we aimed to compare the on/off ratio properties of DIPA-NcoumA with the former probe NcoumA, to evaluate their suitability as fluorogenic probes. The excitation and emission wavelengths chosen for the measurements were 342 nm and 440 nm, respectively, for the free amine Ncoum (considered as a control), and the caged compounds NcoumA and DIPA-NcoumA (Fig. 3b). Among them, DIPA-NcoumA exhibited an impressive on/off ratio of 20 in aqueous solution. This ratio was four times higher than that of the first-generation probe NcoumA. This significant improvement in the on/off ratio has the potential to reduce the background noise and enhance the sensitivity of fluorescence measurements, making DIPA-NcoumA a promising candidate for fluorogenic applications. The UV absorption spectra of both NcoumA and DIPA-NcoumA were then examined at different pH levels, revealing distinct results (Fig. 3c and d). NcoumA displayed similar absorption spectra within the pH range from 4 to 11. In contrast, the absorption of DIPA-NcoumA exhibited a red shift when exposed to basic buffers. We postulated that the deprotonation state (pH above 7) of the diisopropylamino group might influence the absorption properties of the compound. It is also important to mention that DIPA-NcoumA is more water-soluble than NcoumA, as only 1% of DMSO was sufficient to dissolve the new probe.

**Fig. 4:**
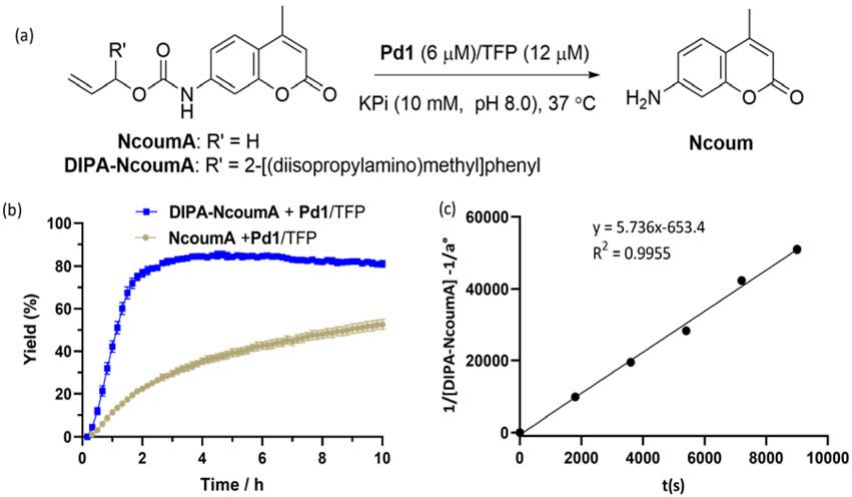
(a) Pd1/TFP-catalyzed deprotection of NcoumA and DIPA-NcoumA in 10 mM KPi (pH 8.0). (b) Comparison of reaction rates between DIPA-NcoumA and NcoumA. Conditions: NcoumA (5% DMSO in KPi, 1.0 equiv., 6 μM) or DIPA-NcoumA (1% DMSO in KPi, 1.0 equiv., 6 μM), Pd1 (6 μM, 1 equiv.) and TFP (12 μM, 2 equiv.) in KPi (10 mM, pH 8.0) at 37 °C. Error bars: ± SD from *n* = 3. (c) Determination of the kinetic constant for the reaction between DIPA-NcoumA and Pd1/TFP. The mathematical reasoning is provided in Fig. S8 and S9.†

Then, our next objective was to evaluate the release kinetics of the caged probes NcoumA and DIPA-NcoumA under conditions enabling Tsuji–Trost deallylation, using complex Pd1, which was previously developed in our group, along with tri(2-furyl)phosphine (TFP) as a ligand (Fig. 4a).^34^ By employing fluorescence emission of the released Ncoum molecule as a monitoring tool, it became apparent that NcoumA couldn't reach a plateau after incubation for 10 h, while DIPA-NcoumA achieved a plateau in only 3 h (Fig. 4b). Using equimolar amounts of DIPA-NcoumA and Pd1/TFP, high-performance liquid chromatography (HPLC) experiments allowed us to determine that the second-order rate constant *k*_2_ of the deallylation reaction was 5.74 ± 0.51 M^−1^ s^−1^ in KPi at pH 8.0 (Fig. 4c). Under identical conditions, *k*_2_ of NcoumA deallylation was only 0.42 ± 0.04 M^−1^ s^−1^ (Fig. S10†). The turnover number (TON) and the turnover frequency (TOF) were also calculated to assess the catalytic activity.^68^ When only 4% of Pd1/TFP were used to deprotect DIPA-NcoumA, Ncoum was obtained in a 6% yield after 30 min, giving a TON of 1.5 and a TOF of 3.0 h^−1^. However, when NcoumA was subjected to the same catalytic conditions, only 2.7% of Ncoum was detected, resulting in a TON of 0.7 and a TOF of 1.4 h^−1^ (Fig. S11†). These results suggested that DIPA-NcoumA can be efficiently deprotected by the Pd1/TFP catalyst under conditions close to physiological ones (Table 1).

**Table 1:** Summary of the kinetic parameters for deallylation of NcoumA and DIPA-NcoumA

Probe	TON	TOF (h^−1^)	*k*_2_ (M^−1^ s^−1^)
NcoumA	0.7	1.4	0.42 ± 0.04
DIPA-NcoumA	1.5	3.0	5.74 ± 0.51

**Fig. 5:**
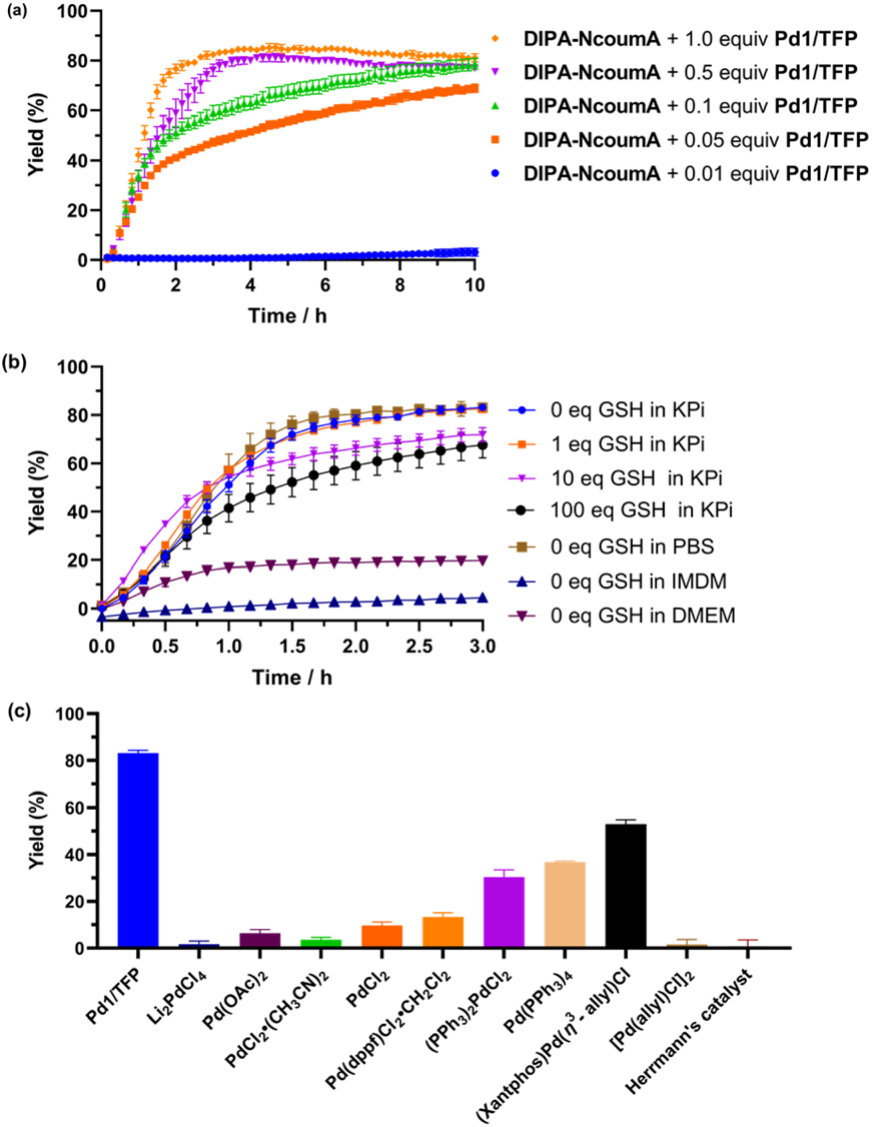
(a) Assessment of the impact of Pd1/TFP catalyst loading on the Tsuji–Trost deallylation reaction. (b) Evaluation of the influence of GSH and various buffers on the reaction. (c) Screening of various palladium complexes for catalytic deallylation, with fluorescence intensity measured after 3 h. The kinetic curves over 10 h were provided in Fig. S13.† Conditions: DIPA-NcoumA (6 μM, 1.0 equiv.), Pd1 (6 μM, 1 equiv.)/TFP (12 μM, 2 equiv.), or other palladium complexes (6 μM, 1.0 equiv.) in 1% DMSO in KPi (10 mM, pH 8.0) at 37 °C. Error bars: ± SD from *n* = 3.

We then proceeded to investigate the genuine catalytic conditions and the limits of the Pd/probe ratio. At a ratio of 0.01, no significant fluorescence was observed. However, when the catalyst loading was increased to above 0.05, a robust fluorescence signal emerged, which was indicative of potent catalytic activity (Fig. 5a). We also assessed the influence of external nucleophiles, such as glutathione (GSH),^69^ and common buffers such as Phosphate-Buffered Saline (PBS), Dulbecco's Modified Eagle Medium (DMEM), and Iscove's Modified Dulbecco's Medium (IMDM).^70^ Elevating the GSH concentration resulted in a slowdown in the Tsuji–Trost deallylation process, and we suspected a possible partial deactivation of the catalyst when high concentrations of GSH (>10 equivalents) were present in the reaction media. Nevertheless, a significant part of the reactivity remained, even with 100 equivalents of GSH. When the catalyst was incubated with DMEM and IMDM, most of the catalytic activity was lost, indicating a possible deactivation of the catalyst by some components of those cell culture media (Fig. 5b). However, we found that using such media for cell culture before incubation with the probe and the catalyst did not prevent the catalytic deallylation reaction inside the cells (*vide infra*). We also tested the cleavage of the DIPA-NcoumA probe using various commercial palladium sources, including Pd(0), Pd(ii), monomers, and dimers (Fig. 5c). Our screening began with common Pd(ii) salts, such as Li_2_PdCl_4_, Pd(OAc)_2_, PdCl_2_·(CH_3_CN)_2_, and PdCl_2_. These palladium sources showed relatively low catalytic efficiency in the deallylation of DIPA-NcoumA. Notably, PdCl_2_ exhibited the best performance among these salts. The use of the bidentate ligand 1,1′-bis(diphenylphosphino)ferrocene (dppf) induced a slight increase in fluorescence intensity, which meant a more efficient deallylation reaction. The employment of Pd(PPh_3_)_2_Cl_2_ led to a significant improvement in the release process. To explain this result, we hypothesized that phosphine ligands could promote the activation of the precatalytic Pd(ii) species through base-mediated reduction, thus forming the active Pd(0) species.^71,72^ Using Pd(PPh_3_)_4_ as a Pd(0) source gave a similar reaction rate. Another well-known palladium precatalyst, (Xantphos)Pd(η^3^-allyl)Cl,^73^ exhibited a rapid release within the initial 30 minutes but reached a lower plateau than that observed using Pd1/TFP (Fig. S13†). This observation led us to speculate that the active Pd(0) complex generated by the (Xantphos)Pd(η^3^-allyl)Cl precatalyst might be less stable compared to that generated by the Pd1/TFP catalytic system. Finally, we explored the activities of two dimers, namely [Pd(allyl)Cl]_2_ and Herrmann's catalyst.^74^ Both dimers produced negative results, suggesting a lack of active Pd(0) generation or a lack of Pd(0) stability under the studied conditions, as no stabilizing ligand was employed in those cases.

**Fig. 6:**
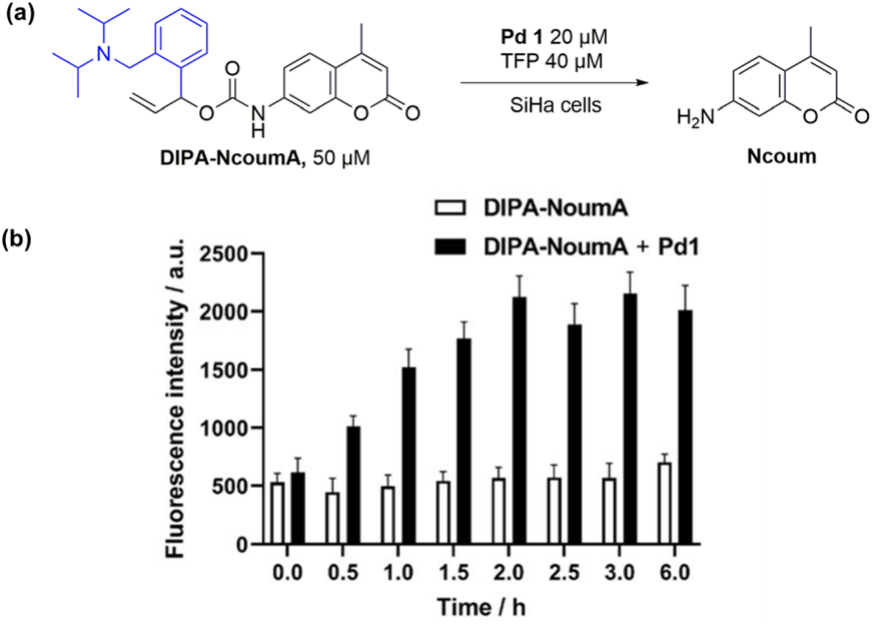
(a) Pd1/TFP-catalyzed transformation of DIPA-NcoumA into Ncoum in SiHa cells. (b) Bar diagram representation of fluorescence intensity obtained at different time points in SiHa cells. Conditions: DIPA-NcoumA (50 μM), incubation for 18 h, washing with PBS three times, addition of Pd1 (20 μM) and TFP (40 μM), then fluorescence measurements (*λ*_ex_ = 342 nm, *λ*_em_ = 440 nm) at 37 °C every 0.5 h. Error bars: ± SD from *n* = 3.

The newly developed caged probe DIPA-NcoumA also exhibited positive release behaviors in SiHa cells (Fig. 6). The fluorescence intensity of the negative control, which was not treated with Pd1/TFP, remained unchanged during 6 h of incubation in SiHa cells. However, when Pd1/TFP were used, a significant increase in fluorescence intensity was observed, reaching a plateau after 2 h. This demonstrated the successful activation and cleavage of the caged probe in living cells, further validating its utility for cellular imaging applications.

**Scheme 3:**
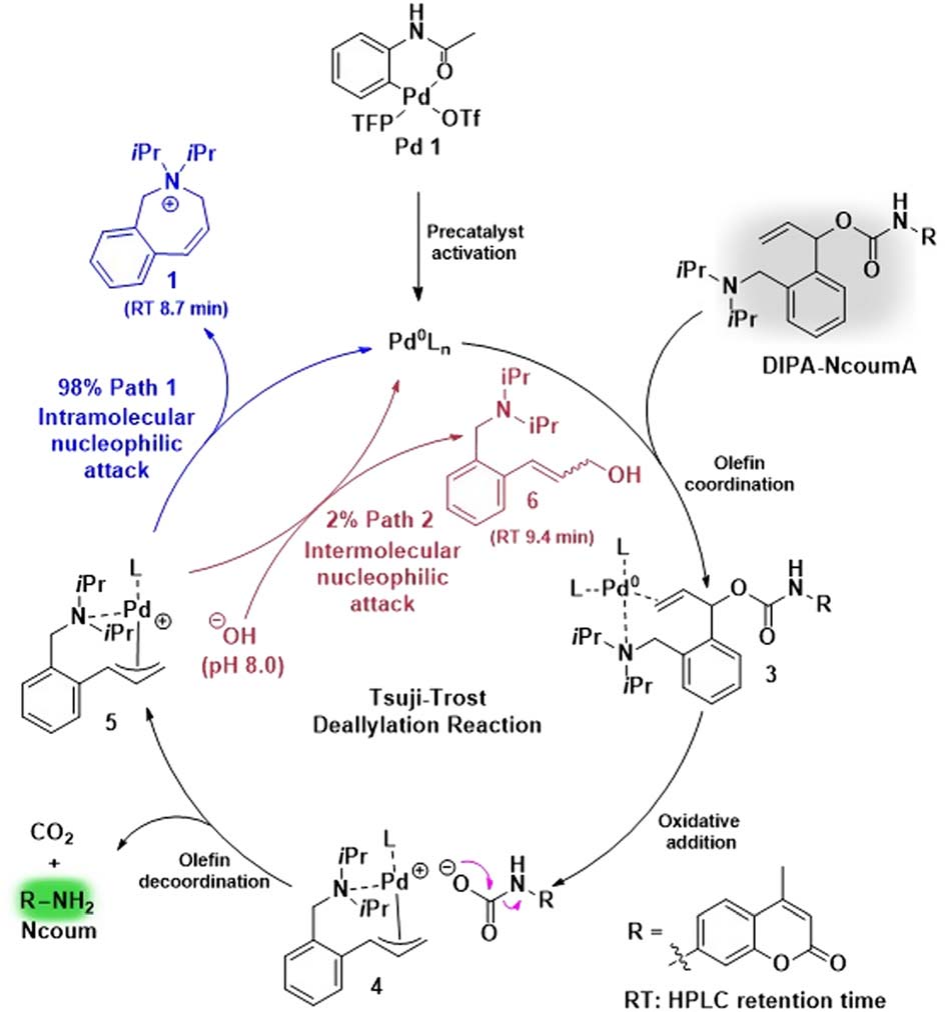
Proposed mechanism for the Tsuji–Trost deallylation of DIPA-NcoumA using Pd1/TFP as a catalyst. Compounds 1 and 6 were detected by LC-MS (1 : 6 = 98 : 2).

Our findings suggested that the diisopropylamino group in DIPA-NcoumA was responsible for an increase in the deallylation kinetics. In an attempt to rationalize these results, we proposed a catalytic cycle illustrating the likely influence of the diisopropylamino group on the Tsuji–Trost reaction (Scheme 3). First, this group could act as a base, promoting the reduction of the precatalyst Pd1*via* a Heck-type reaction involving the allyl moiety of the Alloc-type protecting group.^34,75^ The active Pd(0) species thus released could then catalyze the Tsuji–Trost deallylation reaction. Secondly, the amine function could make the palladium centre more nucleophilic, leading to the acceleration of the oxidative addition step. Furthermore, two distinct pathways were identified regarding the trapping of the π-allylpalladium intermediate. In the first path, the diisopropylamino group, acting as an internal nucleophile, could react with intermediate 5 to yield both compound 1 and Ncoum. The second path comes into play when water molecules serve as external nucleophiles, leading to the formation of allylic alcohol 6 alongside Ncoum. LC-MS analysis unveiled the generation of compounds 1 and 6 in a ratio of about 49 : 1, highlighting the predominance of the first path in the release process (Fig. S12†). The structure of the key cyclic compound 1 was confirmed by ^1^H-NMR and ^1^H–^1^H COSY experiments (Fig. S6 and S7†). In summary, the internal diisopropylamino moiety, despite its steric hindrance, is a much better nucleophile towards the π-allylpalladium intermediate than water molecules, *i.e.*, the solvent. Such a result was anticipated because intramolecular reactions are presumed to be faster than intermolecular ones,^64^ and water is a relatively weak nucleophile in Tsuji–Trost reactions.^76^ Thus, the intramolecular nature of the π-allylpalladium capture step is likely the primary reason for the improvement in deallylation kinetics compared to the former probe NcoumA, which does not include an internal nucleophilic function.

## Conclusions

We studied three generations of coumarin-derived caged probes bearing Alloc-type protecting groups. Through the intrinsic instability of the second-generation probe DMA-NcoumA, we deduced that the additional dimethylamino group was so nucleophile that it could spontaneously attack the terminal carbon–carbon double bond of the allyl carbamate moiety. The third-generation probe DIPA-NcoumA incorporating a sterically hindered diisopropylamino group addressed this issue. In other words, enhancing the steric hindrance around the tertiary amine allowed us to achieve a good compromise in nucleophilicity: this prevented the probe from spontaneously fragmenting under physiological conditions while maintaining the ability of this amine to intramolecularly trap a π-allylpalladium electrophile.

From the first-generation probe NcoumA to the third-generation probe DIPA-NcoumA, the on/off ratio increased four-fold. Meanwhile, the water solubility of DIPA-NcoumA was better than that of NcoumA. Subsequently, we chose Pd1/TFP to assess the reaction kinetics of the uncaging process. In this context, the third-generation probe showed a higher release rate than the first-generation one in physiological conditions, which was probably because its additional diisopropylamino group can capture the π-allylpalladium intermediate in an intramolecular fashion, as demonstrated through LC-MS analysis. Moreover, Pd1/TFP successfully promoted Tsuji–Trost deallylation of DIPA-NcoumA in living cells. Those exciting results proved that the newly designed compound DIPA-NcoumA was a suitable and promising probe in bioorthogonal uncaging chemistry. Moreover, we anticipate that the diisopropylaminobenzyl-modified Alloc protecting group, which was used in this study to protect 7-amino-4-methylcoumarin, could be employed to mask other amines of interest, thereby creating new caged fluorescent probes or even new prodrugs. Indeed, this protecting group is relatively straightforward to install from a synthetic point of view (4 steps), stable in aqueous environment, and above all, easily removable through palladium-catalyzed deallylation.

## Author contributions

Yonghua Tan: methodology, data curation, formal analysis, investigation, writing-original draft. François Pierrard: methodology, data curation, formal analysis, investigation. Raphaël Frédérick: supervision, methodology, visualization, writing –review & editing. Olivier Riant: supervision, methodology, visualization, writing – review & editing.

## Conflicts of interest

There are no conflicts to declare.

## Supplementary Material

RA-014-D3RA08938J-s001
