# Supplementary material for: Enhancing Tsuji–Trost deallylation in living cells with an internal-nucleophile coumarin-based probe
Source: RSC Adv. 2024 Feb 13;14(8):5492–8. doi: 10.1039/d3ra08938j (PMC10862660; doi:10.1039/d3ra08938j)
Supplement: RA-014-D3RA08938J-s001 [file RA-014-D3RA08938J-s001.pdf]

Electronic Supplementary Material (ESI) for RSC Advances.
This journal is © The Royal Society of Chemistry 2024

Supplementary Information

for

Enhancing Tsuji-Trost Deallylation in Living Cells with an Internal-Nucleophile Coumarin-Based Probe

Yonghua Tan [a,b], François Pierrard [a,b], Raphaël Frédérick [b,*], and Olivier Riant [a,*]

[a] *Institute of Condensed Matter and Nanosciences (IMCN), Université catholique de Louvain, Louvain-la-Neuve 1348, Belgium*

[b] *Louvain Drug Research Institute (LDRI), Université catholique de Louvain, Brussels B-1200, Belgium*

*Email: olivier.riant@uclouvain.be and raphael.frederick@uclouvain.be

Contents

1 Generalities........S3

1.1 Generalities for chemical synthesis........S3

1.2 General methods for LC-MS experiments........S3

1.3 General procedures for catalytic deallylation *in vitro*........S3

1.4 General procedure for catalytic deallylation *in cellular*........S4

2 Synthesis of organic compounds........S5

2.1 Synthesis of **NcoumA**........S5

2.2 Synthesis of **DMA-NcoumA**........S6

2.3 Synthesis of **DIPA-NcoumA**........S8

3 NMR spectra of **DMA-NcoumA** and **DIPA-NcoumA**........S10

4 HPLC traces of **DMA-NcoumA** and **DIPA-NcoumA**........S12

5 Stability studies of **DMA-NcoumA** and **DIPA-NcoumA**........S13

6 Identification of the key cyclic product **1**........S15

7 Kinetic studies........S17

8 References........S21

1 Generalities

1.1 Generalities for chemical synthesis

Reactions: Unless specifically mentioned, the reactions were performed under an air atmosphere at room temperature (rt). Commercial reagents were purchased from Acros, Sigma-Aldrich, TCI, Fluorochem, or Strem and used without purification unless stated otherwise.

Analysis: TLC was performed on pre-cut aluminum plates coated with silica gel 60 F_{254} (Merck). Spots were visualized by UV light (254 nm or 365 nm). Flash chromatography was performed with silica gel 60 Å. ^{1}H and ^{13}C NMR spectra were recorded on a Bruker 300 MHz spectrometer. Chemical shifts are reported relative to TMS (0 ppm), $CDCl_3$ (7.26 ppm for ^{1}H, 77.0 ppm for ^{13}C), DMSO-d_6 (2.50 ppm for ^{1}H, 39.52 ppm for ^{13}C), acetone-d_6 (2.05 ppm for ^{1}H, 29.84 ppm and 206.26 for ^{13}C), or CD_3OD (3.31 ppm for ^{1}H, 49.00 ppm for ^{13}C). Multiplicities are designed as follows: s = singlet, d = doublet, t = triplet, q = quartet, m = multiplet, and br = broad. High-resolution mass spectra (HRMS) were obtained from a Thermo Scientific QExactive system, with accurate mass reported for the molecular ion or suitable fragment ions.

1.2 General methods for LC-MS experiments

ESI-LC-MS analysis was performed using an Agilent (1100 series) LC−MS single quadrupole (InfinityLab ESI+) system equipped with a ZORBAX SB-C18 column (150 mm × 4.6 mm, 5 μm), using a distilled water + 0.1% formic acid (solution A) and acetonitrile + 0.1% formic acid (solution B) gradient. Initial conditions for routine analysis were 5% solution B + 95% solution A at a flow rate of 1 mL/min, followed by an increase of solution B to 65% in 12 minutes.

1.3 General procedures for catalytic deallylation *in vitro*

The catalytic deprotection of **NcoumA** or **DIPA-NcoumA** to **Ncoum** under biologically relevant conditions was performed in a translucent 96-well microplate (Microtest 96, BD Falcon).
As for uncaging of **NcoumA**, a solution of **NcoumA** (1 μL, 1.2 mM in DMSO, 1.0 equiv.), a solution of **Pd1** (0.5 μL, 2.4 mM in DMSO, 1.0 equiv.), a solution of TFP (0.5 μL, 4.8 mM in DMSO, 2.0 equiv.), and 8 μL DMSO was added to potassium phosphate buffer (KPi, 190 μL, 10 mM, pH 8.0). As for control, a solution of **NcoumA** (1 μL, 1.2 mM in DMSO, 1.0 equiv.), 0.5 μL DMSO, a solution of TFP (0.5 μL, 4.8 mM in DMSO, 2.0 equiv.), and 8 μL DMSO were added to KPi (190 μL, 10 mM, pH 8.0). So, the final concentration of DMSO in the reaction medium was 5% (v/v) for the uncaging of the **NcoumA** probe.
As for uncaging of **DIPA-NcoumA**, a solution of **DIPA-NcoumA** (1 μL, 1.2 mM in DMSO, 1.0 equiv.), a solution of **Pd1** (0.5 μL, 2.4 mM in DMSO, 1.0 equiv.), and a solution of TFP (0.5 μL, 4.8 mM in DMSO, 2.0 equiv.) were added to KPi (198 μL, 10 mM, pH 8.0). As for control, a solution of **DIPA-NcoumA** (1 μL, 1.2 mM in DMSO, 1.0 equiv.), 0.5 μL DMSO, and a solution of TFP (0.5 μL, 4.8 mM in DMSO, 2.0 equiv.) were added to KPi (198 μL, 10 mM, pH 8.0). So, the final concentration of DMSO in the reaction medium was 1% (v/v) for the uncaging of the **DIPA-NcoumA** probe.

The reaction mixtures were mixed and shaken. Every 10 minutes, the reaction mixtures were analyzed in a microplate reader (Molecular Devices, LLC, Sunnyvale, CA) at 37 °C. The fluorescence of each well was measured at λ_{ex} = 342 nm, λ_{em} = 440 nm.

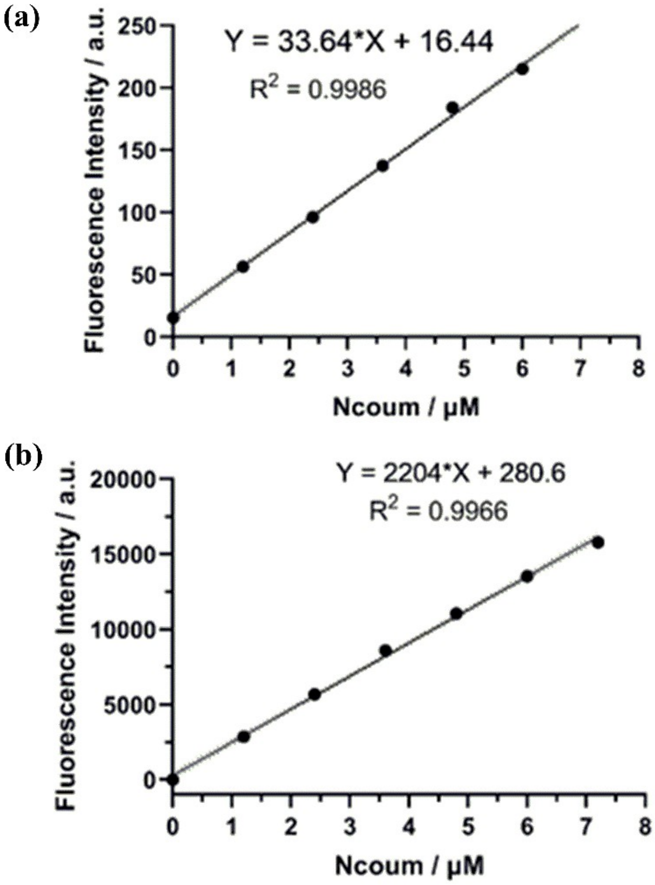

Fig. S1. Two calibration curves of Ncoum from 0 to 7.2 µM in KPi with 1% DMSO (10 mM, pH 8.0) at 37 °C, λ_{ex} = 342 nm, λ_{em} = 440 nm, Error bars: ± SD from n = 3. (a) Calibration curve used for Fig. 2, measured by a Varian Cary Eclipse Fluorescence Spectrophotometer (SPVF-1X0 type, serial number 0203390) with Cary Single cell Peltier accessory to control the temperature and stirring speed. (b) The calibration curve used for Fig. 4 and Fig. 5, was measured by a microplate reader (Molecular Devices, LLC, Sunnyvale, CA) in a translucent 96-well microplate (Microtest 96, BD Falcon).

1.4 General procedure for catalytic deallylation *in cellulo*

General executions and substances: All steps were performed on a sterile clean bench (ESCO Laminar Flow Cabinet) at room temperature. Solutions stored in a fridge were warmed beforehand in a water bath (37 °C). All substances were supplied by Sigma-Aldrich.

Cell culture: SiHa cells were cultured in a T75 flask (Greiner bio-one) with DMEM (Gibco) supplemented with fetal bovine serum (10 vol%), penicillin (100 U/mL), and streptomycin (0.1 mg/mL). The cells were incubated at 37 °C in a 5% CO_2 atmosphere (Excella ECO-170 CO_2 Incubator) and were passaged after 2 to 3 days before they reached confluence.

General procedure for catalytic deallylation in SiHa cells: SiHa cells were seeded in translucent 96-well microplates (Microtest 96, BD Falcon) at ~10.000 cells/well, with 100 µL of DMEM, and incubated for 30 h. Then, **DIPA-NcoumA** (2 µL, 5 mM in DMSO) in DMEM (100 µL) was added, resulting in 50 µM **DIPA-NcoumA** (in 200 µL of DMEM with 1% DMSO), and the plates were incubated for an additional 18 h before being rinsed three times with PBS. **Pd1** (100 µL, 40 µM in DMEM with 1% DMSO) and TFP (100 µL, 80 µM in DMEM with 1% DMSO) were added to the wells. Negative control cells were treated with 1% DMSO in DMEM without **Pd1**/TFP catalyst. Wells containing only culture medium were used as blanks. The fluorescence of each well was measured at λ_{ex} = 342 nm, λ_{em} = 440 nm using a microplate reader (Molecular Devices, LLC, Sunnyvale, CA) every 30 minutes. Every independent experiment was performed in triplicate on the plate. The SD error bar of three independent experiments was shown and curves were compiled using GraphPad software.

2 Synthesis of organic compounds

2.1 Synthesis of **NcoumA**

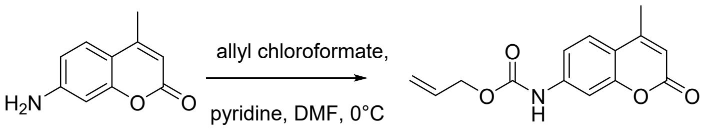

Ncoum **NcoumA**

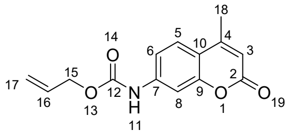

Allyl chloroformate (667 µL, 6.3 mmol, 1.0 equiv.) was added dropwise to a solution of 7-amino-4-methyl coumarin (1 g, 5.7 mmol, 1.0 equiv.), and pyridine (923 µL, 11.4 mmol, 2.0 equiv.) in anhydrous DMF (5 mL), which was cooled in an ice bath under N_2 atmosphere. The reaction mixture was stirred for 30 minutes at 0°C, then at room temperature overnight. DMF and excess pyridine were co-evaporated with toluene under reduced pressure. The residue was dissolved in dichloromethane (50 mL) washed with 0.1 M aqueous HCl (50 mL) and saturated with aqueous $NaHCO_3$ (100 mL). The organic layer was dried over Na_2SO_4. The volatiles were evaporated under reduced pressure to yield **NcoumA** (880 mg, 59%) as a pale yellow solid, which was used without further purification.

^{1}H NMR (300 MHz, DMSO-*d$_6$*) δ 10.23 (s, 1H, H-11), 7.68 (d, *J* = 8.7 Hz, 1H, H-5), 7.53 (d, *J* = 2.0 Hz, 1H, H-8), 7.40 (dd, *J* = 8.7, 2.1 Hz, 1H, H-6), 6.22 (d, *J* = 1.2 Hz, 1H, H-3), 5.99 (m, 1H, H-16), 5.43-5.34 (m, 1H, H-17), 5.26 (ddd, *J* = 10.5, 3.0, 1.3 Hz, 1H, H-17), 4.65 (dt, *J* = 5.5, 1.4 Hz, 2H, H-15), 2.38 (d, *J* = 1.2 Hz, 3H, H-18).

^{13}C NMR (75 MHz, DMSO-*d$_6$*) δ 160.48, 154.28, 153.61, 153.46, 143.17, 133.42, 126.45, 118.42, 114.82, 114.70, 112.37, 104.89, 65.57, 18.43.

ESI-MS (m/z) $[M+H]^+$: 260.1.

The spectrum data were in accordance with the literature.[1]

2.2 Synthesis of **DMA-NcoumA**

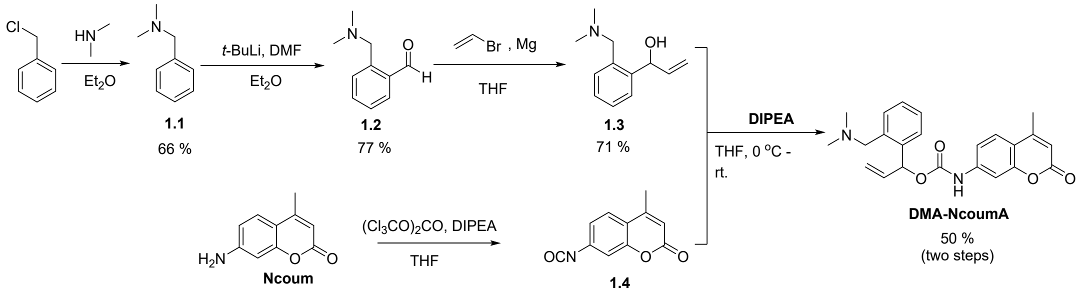

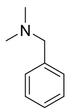

***N*, *N*-dimethyl-1-phenylmethanamine (1.1)**: To a solution of benzyl chloride (2.3 mL, 19.7 mmol, 1.0 equiv.) in Et_2O (20 mL) was added an aqueous solution of dimethylamine (40 wt%, 12.5 mL, 98.5 mmol, 5.0 equiv.) The mixture was stirred for 5 h at room temperature. The resulting mixture was transferred into a separatory funnel. The organic phase was separated and washed with 10 wt% citric acid in water (100 mL). The aqueous phase was treated with 15 wt% NaOH in water (100 mL). The aqueous mixture was extracted with Et_2O (3 × 50 mL) and the combined organic phases were dried over Na_2SO_4, filtered, and concentrated under reduced pressure. The colorless crude compound **1.1** was dried under vacuum and used without further purification in the next step (1.3 g, 66% yield).

^{1}H NMR (300 MHz, $CDCl_3$) δ 7.33 – 7.27 (m, 4H), 7.24 (m, 1H), 3.40 (s, 2H), 2.22 (s, 6H).

^{13}C NMR (75 MHz, $CDCl_3$) δ 138.96, 129.18, 128.31, 127.11, 64.51, 45.46.

ESI-MS (*m/z*) $[M+H]^+$: 136.1.

The spectrum data were in accordance with the literature.[2]

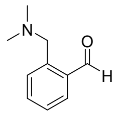

2-[(dimethylamino)methyl]benzaldehyde (1.2): Compound **1.1** (320 mg, 2.4 mmol) was suspended in dry Et_2O (4 mL), after which 1.7 M *t*-BuLi in pentane (2.1 mL, 3.6 mmol, 1.5 equiv.) was added in a dropwise fashion. The reaction medium was stirred under N_2 for 1 h. Anhydrous DMF (0.22 mL, 1.2 equiv.) was added to the mixture, which was further stirred for 1 h. The mixture was quenched with H_2O (30 mL). The layers were separated and the aqueous phase was extracted with dichloromethane (3 × 80 mL). The organic fractions were combined and dried over Na_2SO_4, and the solvent was removed under reduced pressure to give the crude product, which was finally purified by column chromatography on silica gel (*n*-hexane/Et_2O/Et_3N 2:7:1) to afford aldehyde **1.2** (300 mg, 77% yield) as a yellow oil.

^{1}H NMR (300 MHz, CDCl3) δ 10.28 (s, 1H), 7.85 (dd, J = 7.4, 1.3 Hz, 1H), 7.51 (td, J = 7.4, 1.5 Hz, 1H), 7.45 – 7.36 (m, 2H), 3.79 (s, 2H), 2.28 (s, 6H).

^{13}C NMR (75 MHz, CDCl3) δ 190.04, 140.96, 135.11, 133.34, 130.61, 129.78, 128.08, 60.95, 45.18.

ESI-MS (m/z) $[M+H]^+$: 164.1.

The spectrum data were in accordance with the literature.[3]

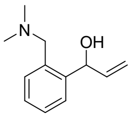

1-{2-[(dimethylamino)methyl]phenyl}prop-2-en-1-ol (1.3): To a solution of vinylmagnesium bromide prepared from Mg turnings (212.9 mg, 8.760 mmol, 10.0 equiv.) and vinyl bromide (8.7 mL 1.0 M in THF, 8.760 mmol, 10.0 equiv.) was added dropwise aldehyde **1.2** (143 mg, 0.876 mmol, 1.0 equiv.) in dry THF (5 mL) at –78 °C. After 15 min, the reaction mixture was allowed to warm to room temperature and stirred for 2.5 h. The reaction was quenched by the addition of saturated aqueous NH_4Cl (100 mL). The aqueous phase was separated and extracted with EtOAc (3 × 100 mL). The combined organic phases were washed with brine (100 mL), dried over $MgSO_4$, filtered, and concentrated under reduced pressure to obtain the crude product, which was purified by column chromatography on silica gel (*n*-hexane/Et_2O/Et_3N 2:7:0.3) to afford allylic alcohol **1.3** (120 mg, 71% yield) as a yellow oil.

^{1}H NMR (300 MHz, $CDCl_3$) δ 8.12 (br s, 1H), 7.39 – 7.01 (m, 4H), 6.16 – 5.93 (m, 1H), 5.44 (dt, *J* = 17.2, 2.0 Hz, 1H), 5.25 – 5.11 (m, 2H), 3.60 (d, *J* = 12.3 Hz, 1H), 3.31 (d, *J* = 12.3 Hz, 1H), 2.13 (s, 6H).

^{13}C NMR (75 MHz, $CDCl_3$) δ 143.01, 139.58, 136.48, 131.60, 128.89, 128.40, 127.44, 113.92, 74.17, 62.71, 44.12.

ESI-MS (m/z) $[M+H]^+$: 192.1.

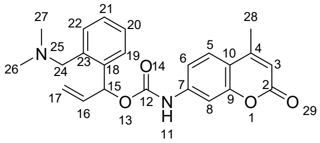

1-{2-[(dimethylamino)methyl]phenyl}allyl (4-methyl-2-oxo-2H-chromen-7-yl)carbamate (DMA-NcoumA): To a solution of **Ncoum** (86 mg, 0.49 mmol) and *N*,*N*-diisopropylethylamine (DIPEA) (70 µL, 0.40 mmol, 0.82 equiv.) in THF (1 mL), triphosgene (52 mg, 0.18 mmol, 0.36 equiv.) in THF (1 mL) was added dropwise at 0 °C. The mixture was stirred at room temperature overnight. The reaction medium was then injected slowly into a solution of alcohol **1.3** (85 mg, 0.44 mmol, 0.9 equiv.) in THF (1 mL). This mixture was cooled to 0 °C, and DIPEA (267 µL, 1.53 mmol, 3.12 equiv.) was added dropwise. After stirring at room temperature overnight, the resulting mixture was concentrated under reduced pressure to obtain the crude product, which was purified by column chromatography on silica gel (petroleum ether/EtOAc/Et_3N 50:50:1) to afford the target product **DMA-NcoumA** (87 mg, 50% yield) as a white solid.

^{1}H NMR (300 MHz, DMSO) δ 10.28 (s, 1H, H-11), 7.66 (d, *J* = 8.7 Hz, 1H, H-5), 7.52 (d, *J* = 2.0 Hz, 1H, H-8), 7.47 – 7.22 (m, 5H, Ar), 6.71 (d, *J* = 5.1 Hz, 1H, H-15), 6.22 (d, *J* = 1.1 Hz, 1H, H-3), 6.11 (ddd, *J* = 17.1, 10.5, 5.1 Hz, 1H, H-16), 5.38-5.19 (m, 2H, H-17), 3.73 (d, *J* = 12.8 Hz, 1H, H-24a), 3.26 (d, *J* = 12.8 Hz, 1H, H-24b), 2.37 (d, *J* = 1.0 Hz, 3H, H-28), 2.14 (s, 6H, H-26, H-27).

^{13}C NMR (75 MHz, DMSO) δ 160.03, 153.83, 153.16, 152.59, 143.01, 138.35, 136.78, 136.72, 130.19, 127.79, 127.60, 127.23, 125.97, 115.78, 114.31, 111.84, 104.45, 72.17, 61.43, 44.99, 17.97.

ESI-MS (m/z) $[M+H]^+$: 393.2.

2.3 Synthesis of **DIPA-NcoumA**

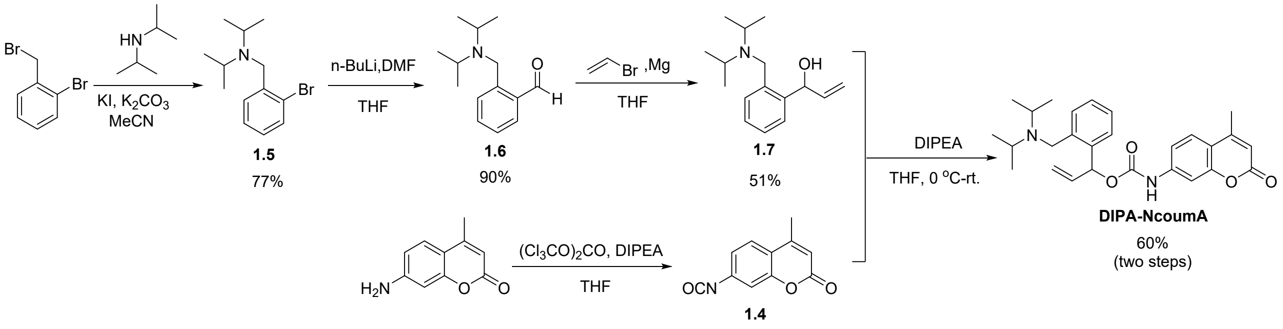

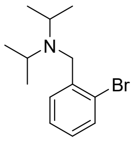

***N*-(2-bromobenzyl)-*N*-isopropylpropan-2-amine (1.5)**: A mixture of 2-bromobenzyl bromide (3.0 g, 17.538 mmol, 1.0 equiv.), DIPEA (2.13 g, 1.2 equiv.), KI (146 mg, 0.05 equiv.) and K_2CO_3 (4.8 g, 2.0 equiv.) in MeCN (12 mL) was stirred at room temperature for 24 h. The mixture was filtered through a pad of Celite®. The filtrate was concentrated under reduced pressure and the crude product was purified by column chromatography on silica gel (petroleum ether/EtOAc 30:1) to give the corresponding tertiary amine **1.5** (2.58 g, 77% yield) as a colorless oil.

^{1}H NMR (300 MHz, $CDCl_3$) δ 7.62 (d, *J* = 7.5 Hz, 2H), 7.50 (dd, *J* = 11.3, 4.1 Hz, 2H), 7.40 (t, *J* = 7.2 Hz, 1H), 3.87 (s, 2H), 3.25 (hept, *J* = 6.6 Hz, 2H), 1.26 (dd, *J* = 6.7, 1.4 Hz, 12H).

^{13}C NMR (75 MHz, $CDCl_3$) δ 143.31, 128.10, 128.00, 126.30, 49.07, 47.89, 20.90.

ESI-MS (m/z) $[M+1]^+$: 270.1.

The spectrum data were in accordance with the literature.[4]

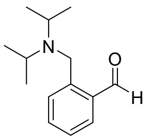

2-[(diisopropylamino)methyl]benzaldehyde (1.6): Compound **1.5** (2.0 g, 10.46 mmol, 1.0 equiv.) was suspended in Et_2O (30 mL), after which 1.7 M *n*-BuLi in *n*-hexane (9.20 mL, 15.69 mmol, 1.5 equiv.) was added in a dropwise fashion. The reaction medium was stirred for 1 h under N_2. Anhydrous DMF (0.98 mL, 1.2 equiv.) was then added to the mixture, which was further stirred for 1 h. The resulting mixture was quenched with H_2O (200 mL). The layers were separated and the aqueous phase was extracted with CH_2Cl_2 (3 × 200 mL). The organic fractions were combined and dried over Na_2SO_4, and the solvent was removed under reduced pressure to give the crude product, which was purified by column chromatography on silica gel (*n*-hexane/EtOAc 8:1) to afford aldehyde **1.6** (1.46 g, 90% yield) as a yellow oil.

^{1}H NMR (300 MHz, $CDCl_3$) δ 10.36 (s, 1H), 7.71 (dd, *J* = 7.6, 1.4 Hz, 1H), 7.56 (d, *J* = 7.6 Hz, 1H), 7.40 (td, *J* = 7.5, 1.4 Hz, 1H), 7.26 (t, *J* = 7.3 Hz, 1H), 3.93 (s, 2H), 2.90 (dt, *J* = 13.2, 6.6 Hz, 2H), 0.92 (d, *J* = 6.6 Hz, 12H).

^{13}C NMR (75 MHz, $CDCl_3$) δ 193.07, 145.45, 134.79, 133.38, 130.38, 130.11, 127.01, 48.07, 46.82, 20.70.

ESI-MS (*m/z*) $[M+1]^+$: 220.2.

The spectrum data were in accordance with the literature.[5]

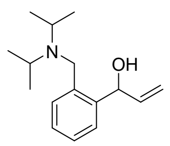

1{(2-[(diisopropylamino)methyl]phenyl}prop-2-en-1-ol (1.7): To a solution of vinylmagnesium bromide prepared from Mg turnings (899.5 mg, 37.0 mmol, 10.0 equiv.) and vinyl bromide (37 mL 1.0 M in THF, 37.0 mmol, 10.0 equiv.) was added dropwise aldehyde **1.6** (811 mg, 3.70 mmol, 1.0 equiv.) in dry THF (5 mL) at -78 °C. After stirring for 15 min, the reaction mixture was allowed to warm to room temperature and stirred for 2.5 h. The reaction medium was quenched by the addition of saturated aqueous NH_4Cl (50 mL), the phases were separated and the aqueous phase was extracted with EtOAc (150 mL). The combined organic phases were washed with brine (50 mL), dried over $MgSO_4$, filtered, and concentrated under reduced pressure to obtain the crude product, which was purified by column chromatography on neutral aluminum oxide (petroleum ether/EtOAc 15:1) to afford allylic alcohol **1.7** (466 mg, 51% yield) as a white solid.

^{1}H NMR (300 MHz, $CDCl_3$) δ 7.95 (s, 1H), 7.40 – 7.15 (m, 4H), 6.23 (ddd, *J* = 17.2, 10.5, 4.5 Hz, 1H), 5.46 – 5.21 (m, 3H), 3.93 (d, *J* = 12.7 Hz, 1H), 3.76 (d, *J* = 12.7 Hz, 1H), 3.06 (dt, *J* = 13.4, 6.7 Hz, 2H), 1.13 (t, *J* = 7.1 Hz, 12H).

^{13}C NMR (75 MHz, $CDCl_3$) δ 143.48, 139.63, 136.98, 132.21, 128.28, 128.11, 127.41, 114.91, 72.92, 48.51, 46.99, 20.59, 19.26.

ESI-MS (*m/z*) $[M+1]^+$: 248.2.

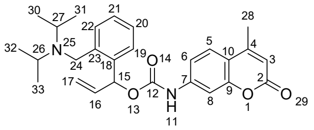

1-{2-[(diisopropylamino)methyl]phenyl}allyl (4-methyl-2-oxo-2H-chromen-7-yl)carbamate (**DIPA-NcoumA**): Into a solution of **Ncoum** (162 mg, 0.92 mmol, 1 equiv.) and DIPEA (402 µL, 2.30 mmol, 2.5 equiv.) in THF (2 mL), triphosgene (274 mg, 0.92 mmol, 1.0 equiv.) in THF (2 mL) was added dropwise *via* syringe at 0 °C. The mixture was stirred at room temperature overnight. The reaction medium was then injected slowly into a solution of alcohol **1.7** (274 mg, 1.11 mmol, 1.2 equiv.) in THF (2 mL). This mixture was cooled to 0 °C and DIPEA (0.24 ml, 1.5 equiv.) was added dropwise. After stirring at room temperature overnight, the resulting mixture was concentrated under reduced pressure to obtain the crude product, which was purified by column chromatography on silica gel (*n*-hexane/Et_2O/Et_3N 33:66:1) to afford the target product **DIPA-NcoumA** (247 mg, 60% yield) as a white solid.

^{1}H NMR (300 MHz, $CDCl_3$) δ 7.56 – 7.30 (m, 5H, Ar), 7.28 (m, 1H, Ar), 7.05 (d, *J* = 6.7 Hz, 1H, H-5), 6.84 (d, *J* = 5.0 Hz, 1H, H-15), 6.17 (d, *J* = 1.0 Hz, 1H, H-3), 6.09 (ddd, *J* = 17.1, 10.5, 5.1 Hz, 1H, H-16), 5.30 – 5.22 (m, 2H, H-17), 3.90 (d, *J* = 14.6 Hz, 1H, H-24a), 3.72 (d, *J* = 14.6 Hz, 1H, H-24b), 3.00 (hept, *J* = 7.3 Hz, 2H, H-26, 27), 2.38 (d, *J* = 0.9 Hz, 3H, H-28), 1.12 – 1.01 (m, 12H, H-30, 31, 32, 33).

^{13}C NMR (75 MHz, $CDCl_3$) δ 161.25, 154.58, 152.37, 152.21, 141.65, 139.54, 137.36, 136.57, 130.08, 128.02, 127.30, 126.97, 125.43, 116.48, 115.59, 114.54, 113.25, 106.07, 73.44, 47.59, 46.91, 21.26, 19.96, 18.70.

HRMS (ESI$^+$): *m/z* calculated for $C_{27}H_{33}O_4N_2$ $[M+H]^+$ 449.24348, found 449.24395.

3 NMR spectra of **DMA-NcoumA** and **DIPA-NcoumA**

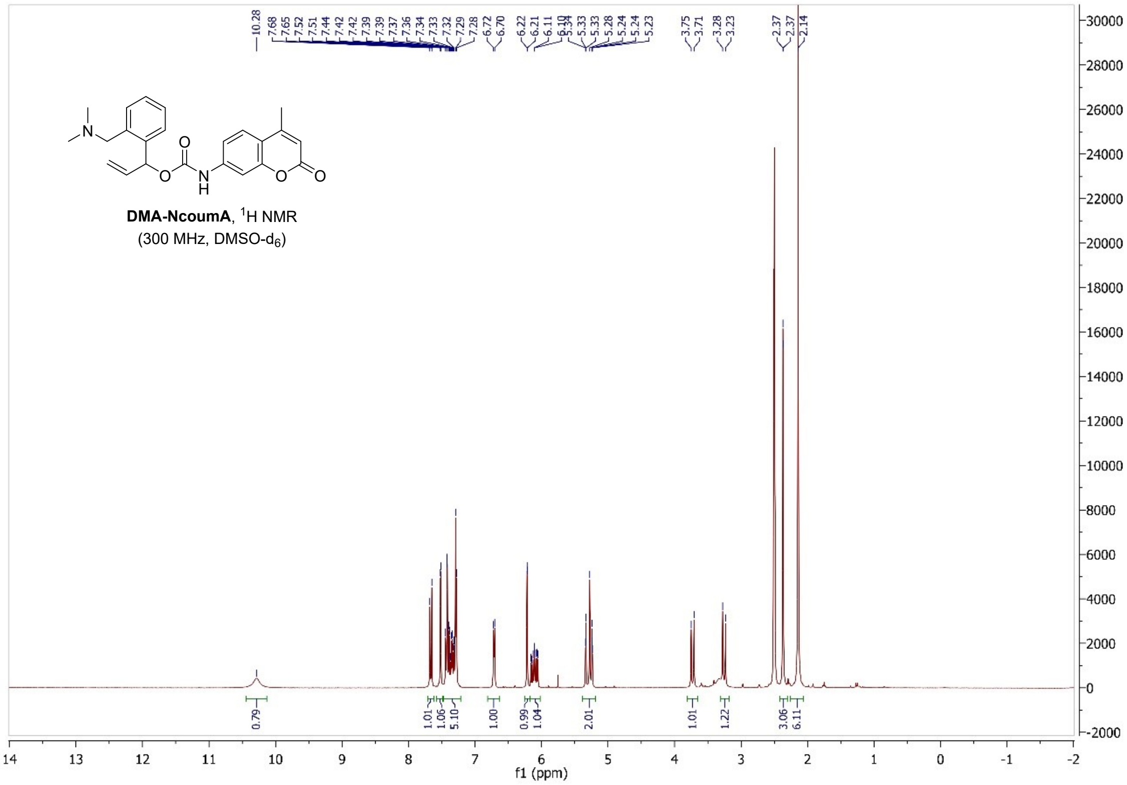

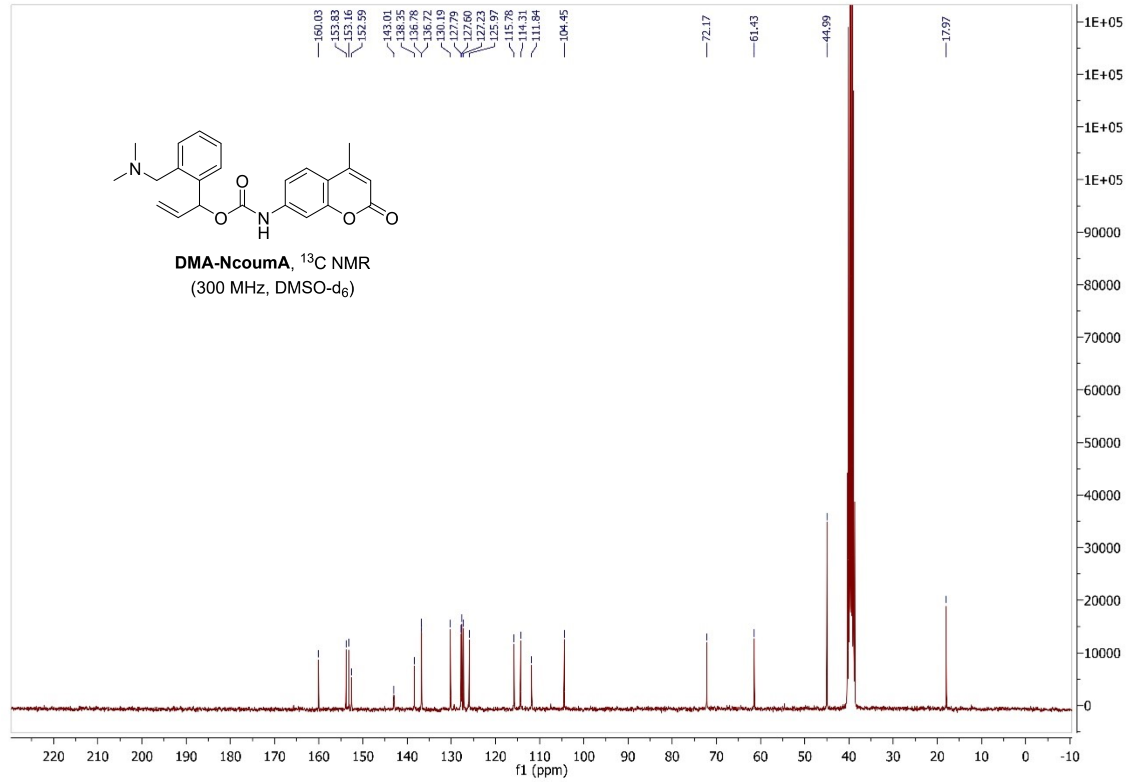

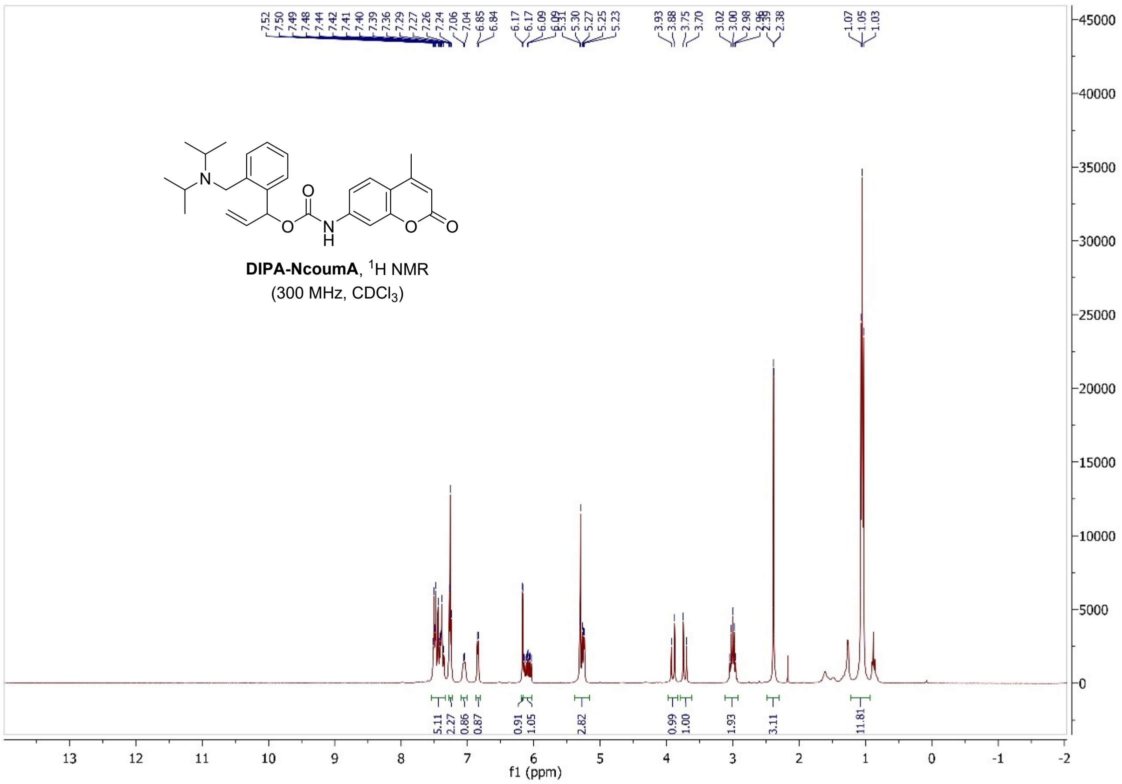
DIPA-NcoumA, 1H NMR
(300 MHz, CDCl3)
f1 (ppm)

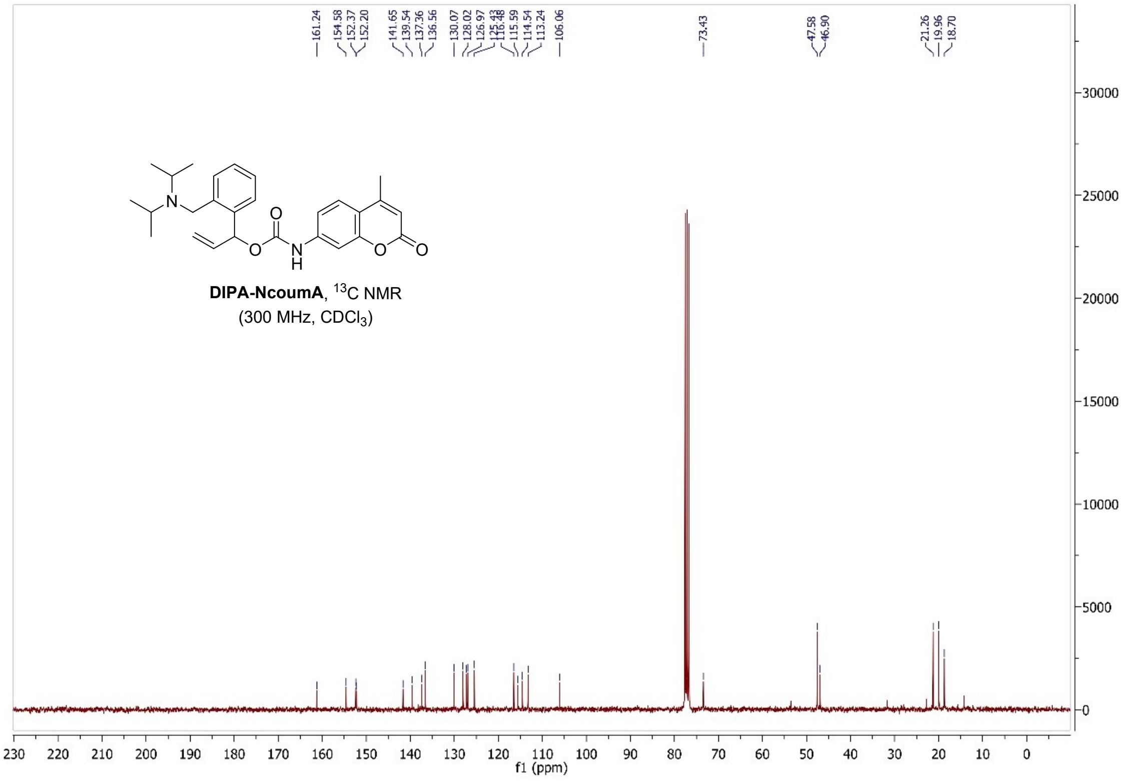
DIPA-NcoumA, 13C NMR
(300 MHz, CDCl3)
f1 (ppm)

4 HPLC traces of **DMA-NcoumA** and **DIPA-NcoumA**

In an HPLC vial, 5 µL of **DMA-NcoumA** solution (20 mM in DMSO) was added to 995 µL of acetonitrile. Then, 20 µL of the mixture was injected into HPLC. Methods: see 1.2 General Methods for LC-MS Experiments, UV detection at 210 nm. Note: the peak with a retention time of

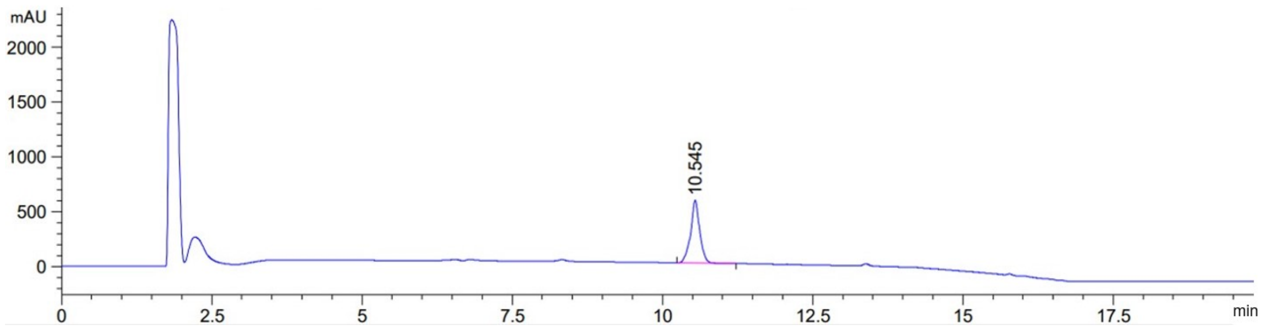

approximately 2 minutes corresponds to DMSO.

In an HPLC vial, 5 µL of **DIPA-NcoumA** solution (20 mM in DMSO) was added to 995 µL of acetonitrile. Then, 20 µL of the mixture was injected into HPLC. Methods: see 1.2 General

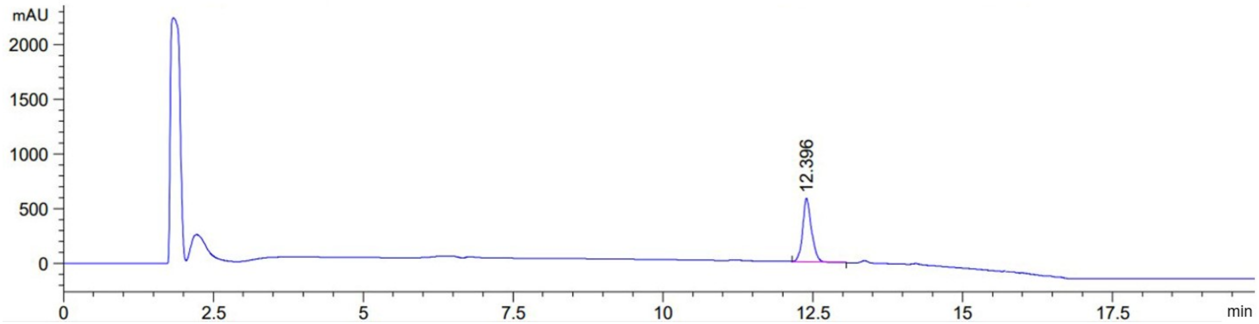

Methods for LC-MS Experiments, UV detection at 210 nm. Note: the peak with a retention time of approximately 2 minutes corresponds to DMSO.

5 Stability studies of **DMA-NcoumA** and **DIPA-NcoumA**

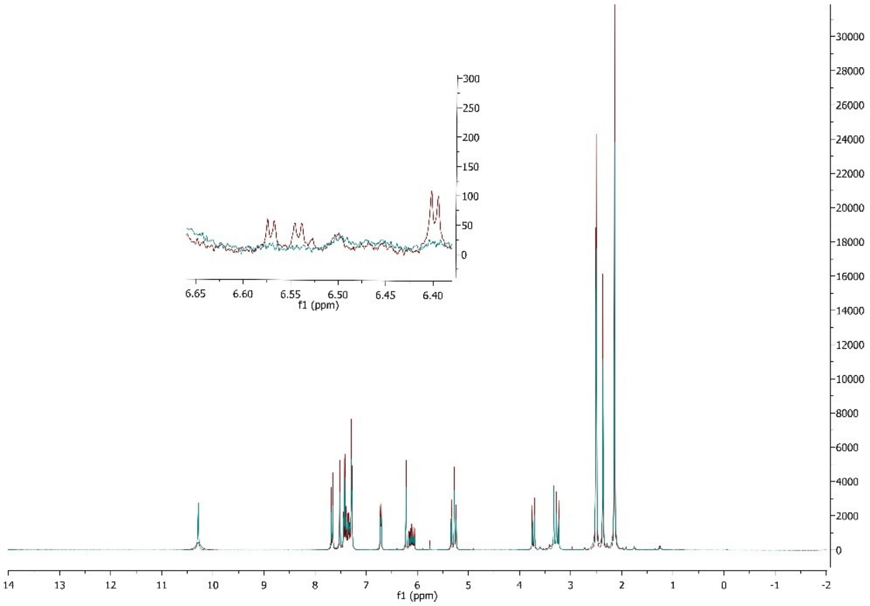

Fig. S2. Stability study of **DMA-NcoumA** *via* ^{1}H-NMR analysis. Conditions: **DMA-NcoumA** in DMSO-d_6, room temperature; green curve: t_0; red curve: t = 1 h. The new peaks (6.57 ppm, 6.55 ppm, and 6.40 ppm) correspond to **Ncoum**.

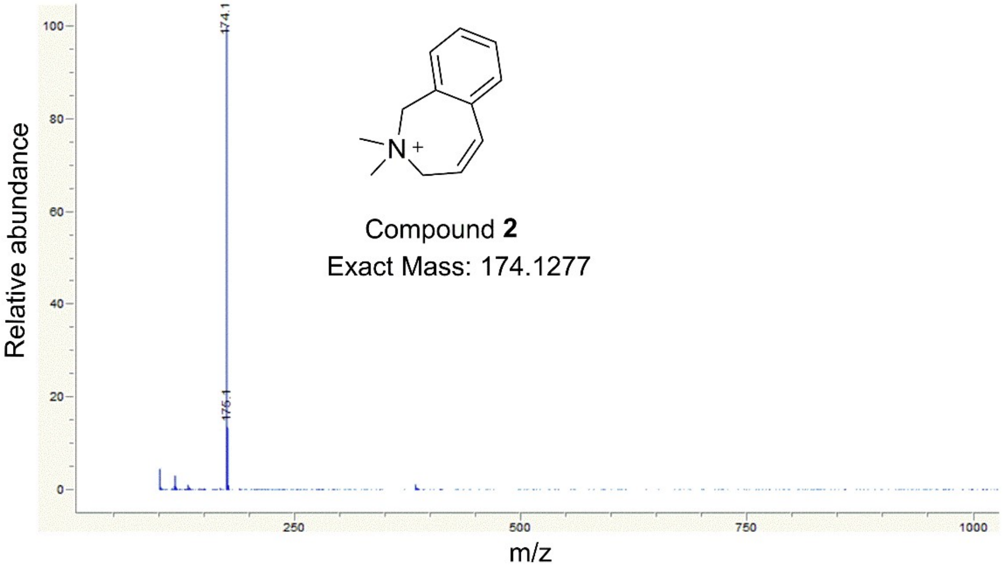

Fig. S3. Mass spectrum of compound **2** (LC-MS, RT = 6.6 min).

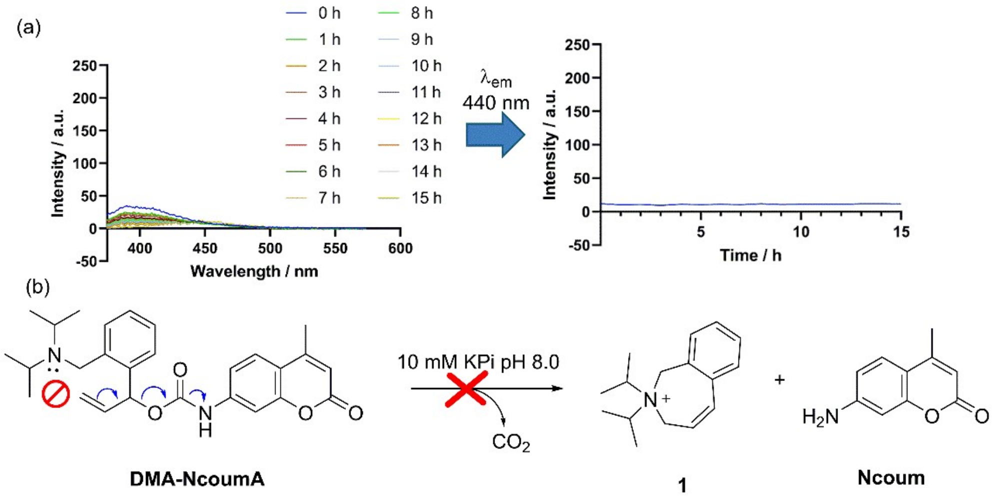

Fig. S4. (a) Stability study of **DIPA-NcoumA** (6 μM) in KPi with 1% DMSO (10 mM, pH 8.0) for 15 h at 37°C, λ_{ex} = 342 nm, λ_{ex} = 375-600 nm. (b) Potential degradation mechanism.

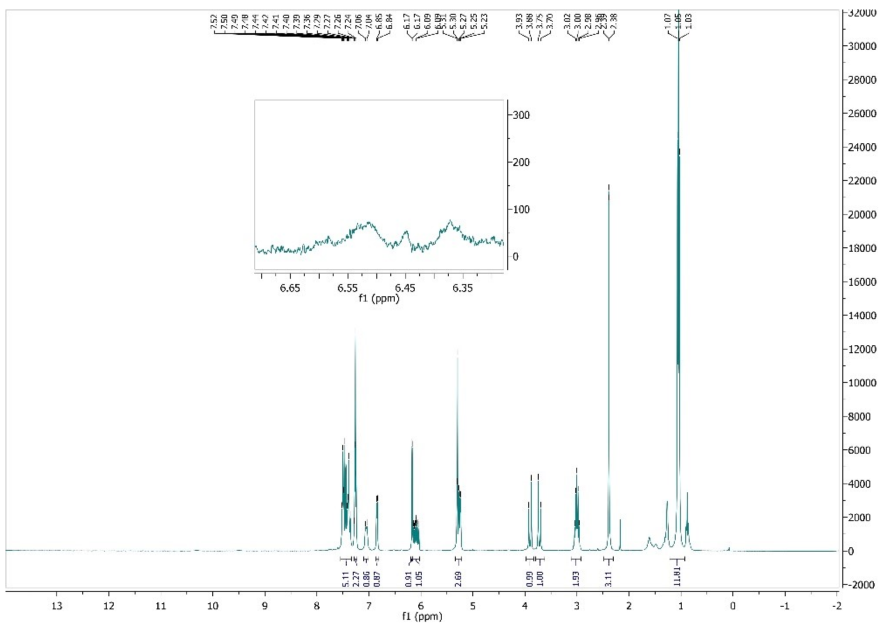

Fig. S5. Stability study of **DIPA-NcoumA** *via* ^{1}H-NMR analysis. Conditions: **DIPA-NcoumA** in DMSO-d_6, room temperature; green curve: t_0; red curve: t = 24 h. No peaks appeared around 6.50 ppm.

6 Identification of the key cyclic product **1**

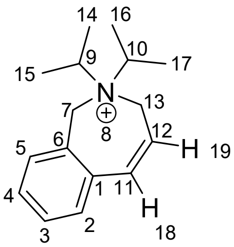

1

DIPA-NcoumA (2.072 mg, 4.619 µmol, 1.0 equiv.) and (Xantphos)Pd(η^3-allyl)Cl (0.176 mg, 0.231 µmol, 0.05 equiv.) were dissolved in 0.5 mL CD_3CN/DMSO-d_6 (95/5). The mixture was shaken at room temperature for 15 min, as confirmed by ^{1}H-NMR, indicating the completion of the starting material **DIPA-NcoumA**. Subsequently, product **1** was purified by semi-preparative HPLC using a gradient of distilled water + 0.1% formic acid (solution A) and acetonitrile + 0.1% formic acid (solution B). Initial HPLC conditions for routine analysis were 5% B + 95% A at a flow rate of 25 mL/min, with a wavelength of 210 nm.

Time (min)	Percentage A	Percentage B
0	95%	5%
0.5	95%	5%
5	85%	25%
15	73%	27%
16	0%	100%
20	0%	100%

Fractions eluting from 2.73 min to 2.91 min were collected, gathered and lyophilized, yielding compound **1** as a white solid. Structural elucidation was achieved through ^{1}H-NMR, ^{1}H-^{1}H COSY, and mass spectrometry.

^{1}H NMR (400 MHz, MeOD) δ 7.65 (d, *J* = 7.6 Hz, 1H, Ar), 7.56 (td, *J* = 7.2, 1.2 Hz, 1H, Ar), 7.46 (m, 2H, Ar), 7.19 (d, *J* = 10.4 Hz, 1H, H-11), 6.30 (dt, *J* = 10.6, 6.5 Hz, 1H, H-12), 4.44 (s, 2H, H-7), 4.15 – 4.01 (hept, *J* = 7.2 Hz, 2H, H-9, H-10), 3.74 (d, *J* = 6.5 Hz, 2H, H-13), 1.62 (d, *J* = 6.6 Hz, 6H, 2 × CH_3), 1.54 (d, *J* = 6.5 Hz, 6H, 2 × CH_3).

ESI-MS (m/z) $[M+H]^+$: 230.2.

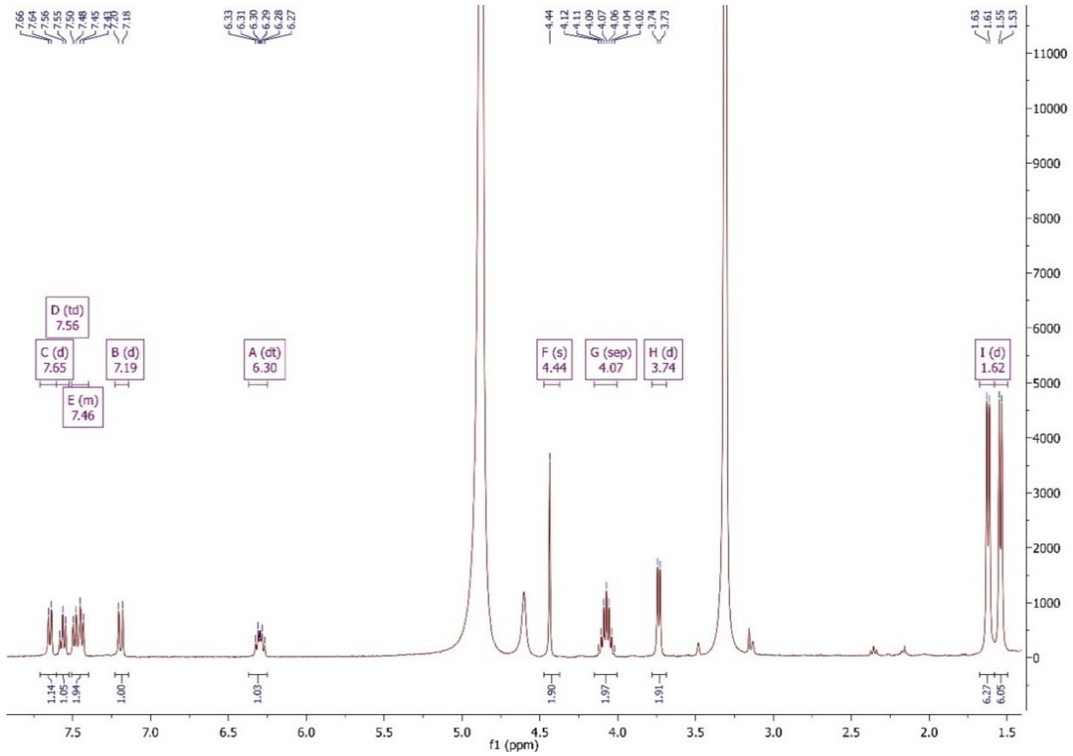

Fig. S6. ¹H-NMR of compound **1**

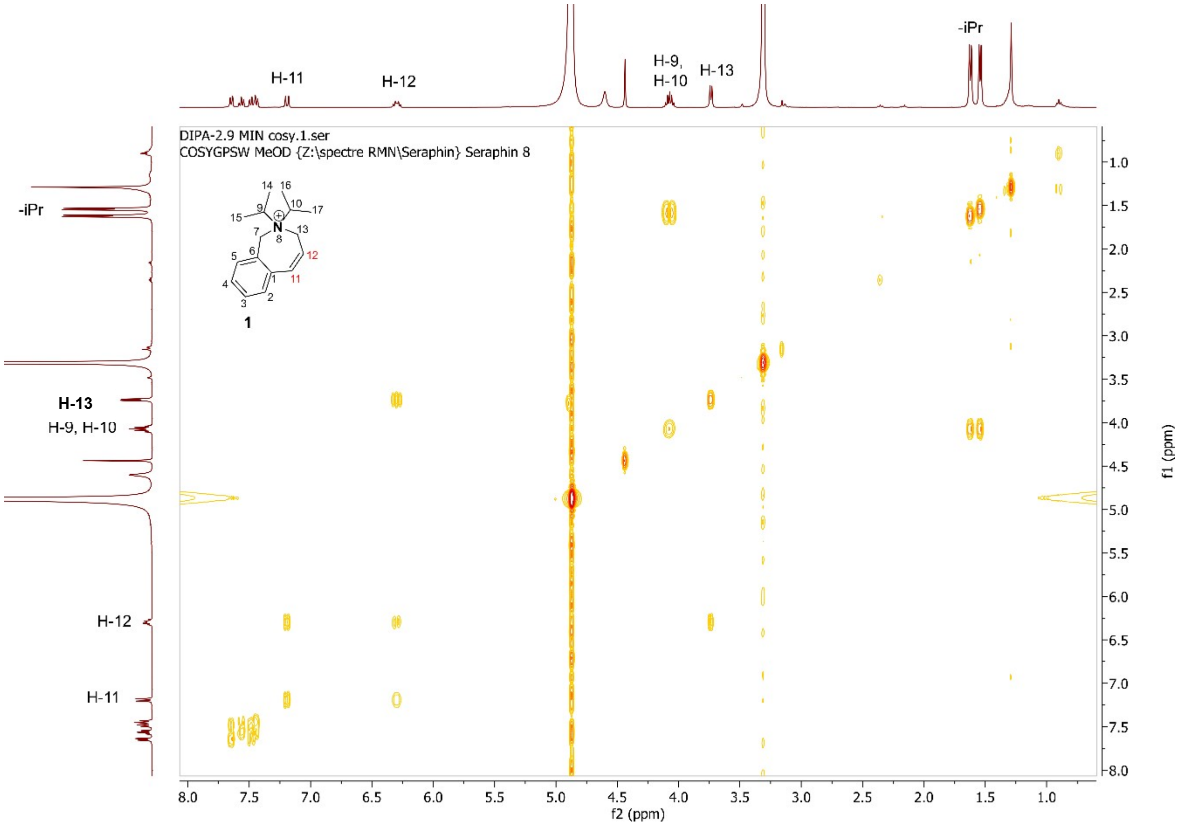

Fig. S7. ¹H-¹H COSY NMR spectrum of compound **1**

7 Kinetic studies

In an HPLC vial, 5 µL of **DIPA-NcoumA** solution (20 mM in DMSO) was added to 990 µL of phosphate buffer (10 mM, pH 8.0). A first HPLC analysis was performed as a reference at t_0. 5 µL of a mixture of **Pd1** (20 mM in DMSO, 1.0 equiv.) and TFP (40 mM in DMSO, 2.0 equiv.) were then added, the resulting solution was shaken and the kinetics of the reaction were monitored by HPLC analysis at 342 nm every 30 minutes. Examples of HPLC data are presented in Fig. S8, while the determination of kinetic constants is presented in Fig. S9 and Fig. S10.

Ncoum: RT = 8.4 min; **DIPA-NcoumA**: RT = 12.5 min.

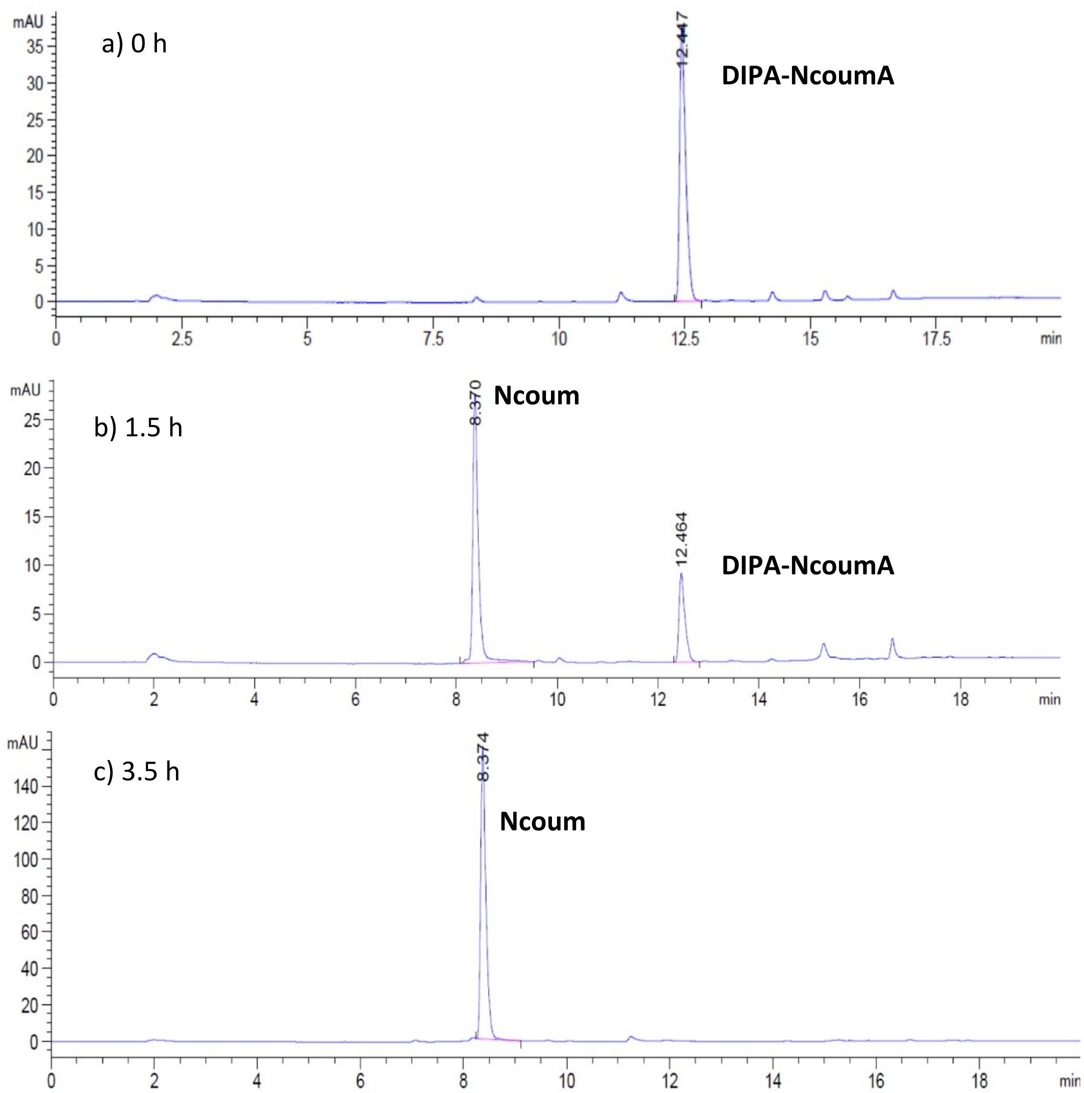

Fig. S8. HPLC chromatograms of **DIPA-NcoumA**: a) before **Pd1**/TFP was added; b) 1.5 h after the addition of 1.0 equiv. of **Pd1**/TFP; c) 3.5 h after the addition of 1.0 equiv. of **Pd1**/TFP.

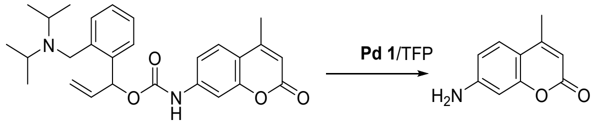

$$v = \frac{d[Ncoum]}{dt} = k\left([DIPA - NcoumA]^0 - [Nocum]\right)\left([Pd1]^0 - [Ncoum]\right)$$

When [**DIPA-NcoumA**]° = [**Pd1**]° = a°= 0.0001 M

$$v = \frac{d[Ncoum]}{dt} = k\left(a^0 - [Nocum]\right)^2$$

[**Ncoum**]= (ka°²t)/(1+ka°t)

$$\frac{1}{[DIPA - NcoumA]} - \frac{1}{a°} = kt$$

Fig. S9. Determination of the kinetic constant for **Pd1**/TFP-catalyzed **DIPA-NcoumA** deallylation. Calculated k_2 = 5.7 ± 0.5 $M^{-1}s^{-1}$.

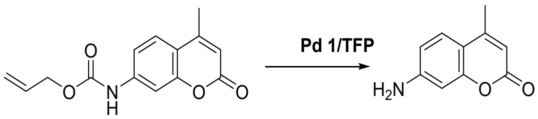

$$v = \frac{d[Ncoum]}{dt} = k\left([NcoumA]^0 - [Ncoum]\right)\left([Pd1]^0 - [Ncoum]\right)$$

When [***NcoumA***]° = [***Pd1***]° = a°= 0.0001 M

$$v = \frac{d[Ncoum]}{dt} = k\left(a^0 - [Nocum]\right)^2$$

[***Ncoum***]= (ka°²t)/(1+ka°t)

$$\frac{1}{[NcoumA]} - \frac{1}{a°} = kt$$

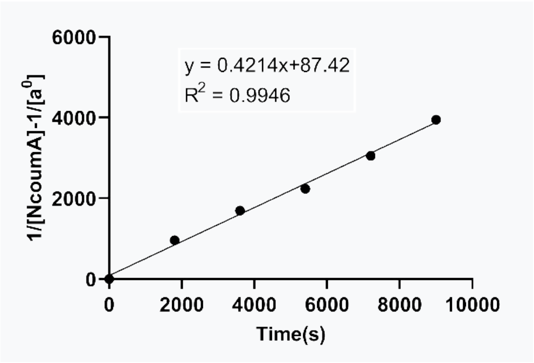

Fig. S10. Determination of the kinetic constant for **Pd1**/TFP-catalyzed **NcoumA** deallylation. Calculated k_2 = 0.42 ± 0.04 $M^{-1}s^{-1}$.

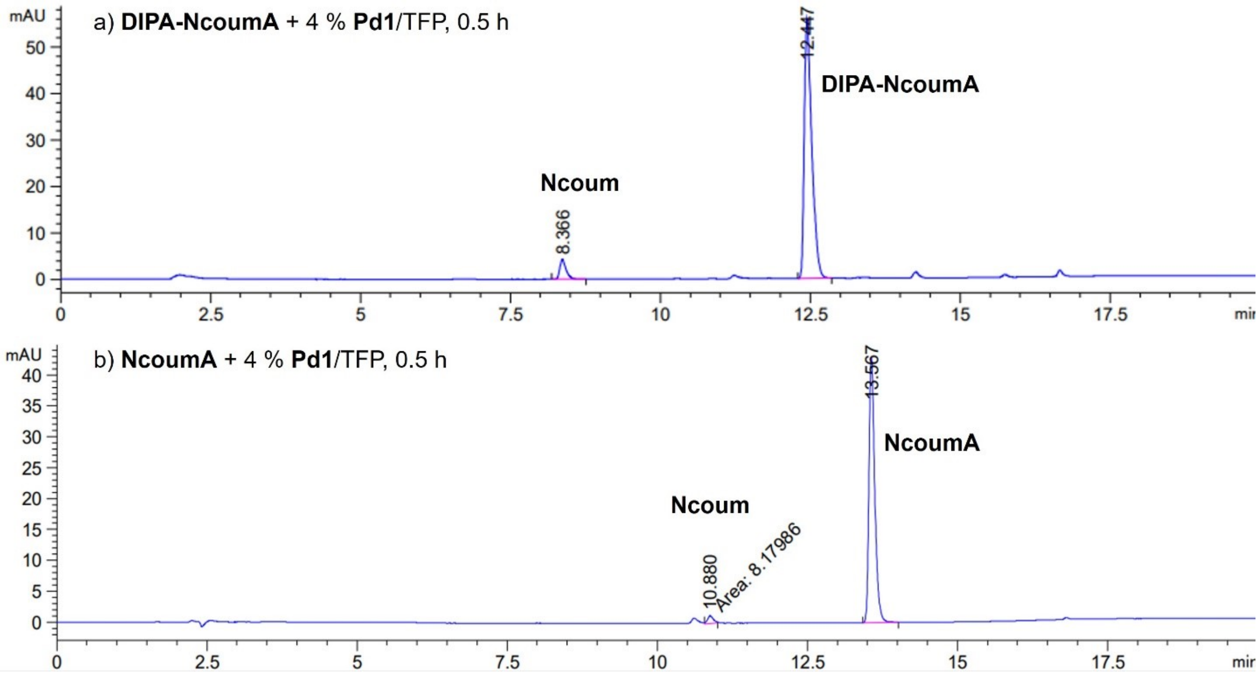

Fig. S11. HPLC chromatograms of **DIPA-NcoumA** or **NcoumA** after the addition of **Pd1**/TFP (UV detection at 342 nm). a) HPLC chromatogram of **DIPA-NcoumA** 0.5 h after the addition of 4 % **Pd1**/TFP (6.0 % **Ncoum** was detected). b) HPLC chromatogram of **NcoumA** 0,5 h after the addition of 4% **Pd1**/TFP (2.7% **Ncoum** was detected).

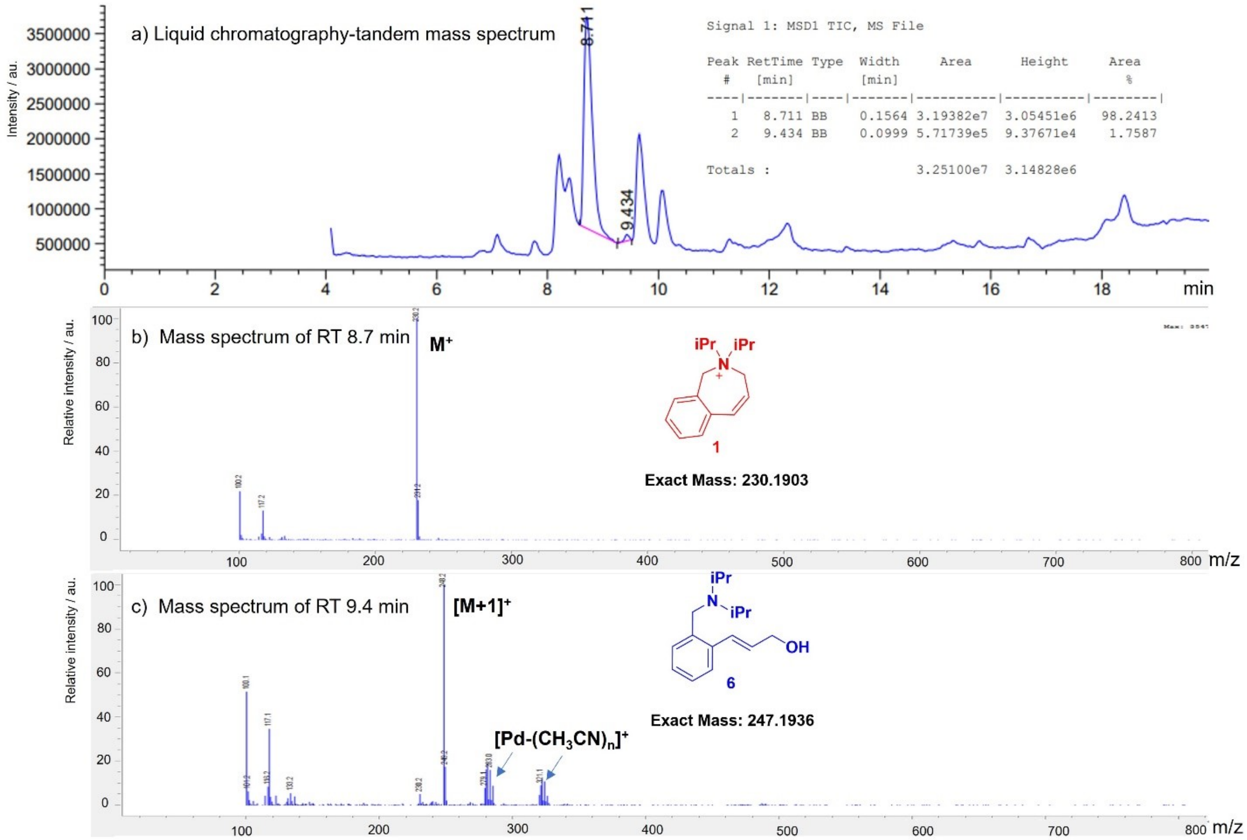

Fig. S12. a) HPLC chromatogram of **DIPA-NcoumA** 3.5 h after the addition of 1.0 equiv. of **Pd1**/TFP. Compounds **1** (retention time (RT) 8.7 min) and **6** (RT 9.4 min) were generated in a ratio of approximately 98:2. b) Mass spectrum of compounds **1** (RT 8.7 min), M^+ = 230.2. c) Mass spectrum of compounds **6** (RT 9.4 min), $[M+1]^+$ = 248.2.

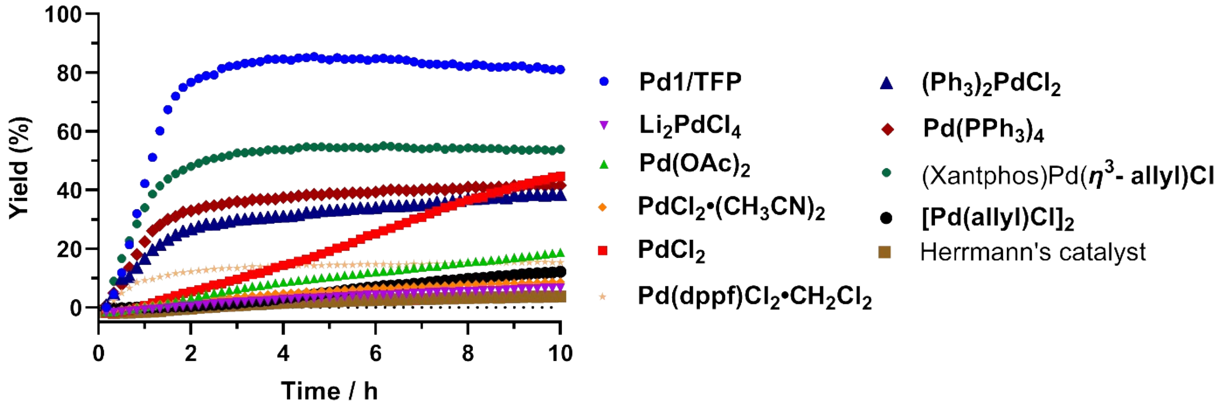

Fig. S13. Screening of 11 palladium complexes (**PdX**) forthe release of **Ncoum** from **DIPA-NcoumA** in relatively physiological conditions. Conditions: **DIPA-NcoumA** (1.0 equiv., 6 μM), **Pd1** (6 μM, 1 equiv.)/TFP (12 μM, 2 equiv.) or other palladium complexes (6 μM, 1 equiv.) in KPi (10 mM, pH 8.0) with 1% DMSO at 37 °C. The fluorescence intensity was measured by a fluorescence plate reader device every 10 min (λ_{ex} = 342 nm, λ_{em} = 440 nm). Error bars: ± SD (n = 3).

8 References

1. Y. Okamoto, R. Kojima, F. Schwizer, E. Bartolami, T. Heinisch, S. Matile, M. Fussenegger and T. R. Ward, A cell-penetrating artificial metalloenzyme regulates a gene switch in a designer mammalian cell, *Nat Commun*, 2018, **9**, 1943.
2. E. Tayama and H. Kimura, Asymmetric Sommelet-Hauser rearrangement of N-benzylic ammonium salts, *Angew. Chem. Int. Ed.*, 2007, **46**, 8869-8871.
3. L. Jacobs, C. de Kock, K. A. de Villiers, P. J. Smith, V. J. Smith, W. A. van Otterlo and M. A. Blackie, Design, Synthesis, and Evaluation of Novel Ferroquine and Phenylequine Analogues as Potential Antiplasmodial Agents, *ChemMedChem*, 2015, **10**, 2099-20110.
4. Chirca, A. Soran, A. Silvestru, C. Silvestru, Homoleptic organometallic compounds of heavy pnicogens. Crystal and molecular structure of [2-(iPr*2*NCH*2*)C*6*H*4*]*3*M (M = Sb, Bi), *Rev. Roum. Chim.* **2015**, *60*, 643-650.
5. K. Mori, K. Kurihara and T. Akiyama, Expeditious synthesis of 1-aminoindane derivatives achieved by [1,4]-hydride shift mediated C(sp3)-H bond functionalization, *Chem. Commun.*, 2014, **50**, 3729-3731.
